# Dual 3’Seq using deepSuperSAGE uncovers transcriptomes of interacting *Salmonella enterica* Typhimurium and human host cells

**DOI:** 10.1186/s12864-015-1489-1

**Published:** 2015-04-19

**Authors:** Fabian Afonso-Grunz, Klaus Hoffmeier, Sören Müller, Alexander J Westermann, Björn Rotter, Jörg Vogel, Peter Winter, Günter Kahl

**Affiliations:** Institute for Molecular BioSciences, Goethe University Frankfurt am Main, Frankfurt am Main, Germany; GenXPro GmbH, Frankfurt Biotechnology Innovation Center (FIZ), Frankfurt am Main, Germany; Institute for Molecular Infection Biology, University of Würzburg, Würzburg, Germany

**Keywords:** Dual 3’Seq, deepSuperSAGE, MACE, Tag-based, Simultaneous, Genome-wide, Gene expression profiling, Host-pathogen interaction, Transcriptome, *Salmonella enterica* Typhimurium strain SL1344

## Abstract

**Background:**

The interaction of eukaryotic host and prokaryotic pathogen cells is linked to specific changes in the cellular proteome, and consequently to infection-related gene expression patterns of the involved cells. To simultaneously assess the transcriptomes of both organisms during their interaction we developed dual 3’Seq, a tag-based sequencing protocol that allows for exact quantification of differentially expressed transcripts in interacting pro- and eukaryotic cells without prior fixation or physical disruption of the interaction.

**Results:**

Human epithelial cells were infected with *Salmonella enterica* Typhimurium as a model system for invasion of the intestinal epithelium, and the transcriptional response of the infected host cells together with the differential expression of invading and intracellular pathogen cells was determined by dual 3’Seq coupled with the next-generation sequencing-based transcriptome profiling technique deepSuperSAGE (deep Serial Analysis of Gene Expression). Annotation to reference transcriptomes comprising the operon structure of the employed *S. enterica* Typhimurium strain allowed for *in silico* separation of the interacting cells including quantification of polycistronic RNAs. Eighty-nine percent of the known loci are found to be transcribed in prokaryotic cells prior or subsequent to infection of the host, while 75% of all protein-coding loci are represented in the polyadenylated transcriptomes of human host cells.

**Conclusions:**

Dual 3’Seq was alternatively coupled to MACE (Massive Analysis of cDNA ends) to assess the advantages and drawbacks of a library preparation procedure that allows for sequencing of longer fragments. Additionally, the identified expression patterns of both organisms were validated by qRT-PCR using three independent biological replicates, which confirmed that *RELB* along with *NFKB1* and *NFKB2* are involved in the initial immune response of epithelial cells after infection with *S. enterica* Typhimurium.

**Electronic supplementary material:**

The online version of this article (doi:10.1186/s12864-015-1489-1) contains supplementary material, which is available to authorized users.

## Background

Interactions between eu- and prokaryotic cells are frequent, multifaceted events ranging from symbiotic synergy such as symbiotic nitrogen fixation in legumes or fermentation by gastrointestinal bacteria to pathogenic interference, for instance, in the course of salmonellosis. This interplay of organisms requires mutual signaling mechanisms and a continuous adaptation of the metabolism of the involved cells to varying environmental conditions. Consequently, programmed expression patterns have to be induced to continuously readjust the proteome and metabolome of both cell types. The characterization of corresponding time-dependent expression patterns allows for a deeper understanding of the underlying molecular processes, and was the focus of numerous studies, but until recently gene expression profiling emphasized either the host cell or the prokaryotic transcriptome [1].

*Salmonella* represents a genus of Gram-negative and facultative anaerobic enterobacteria and is closely related to the genus *Escherichia*. The human-virulent pathogen is a model organism for characterization of host-pathogen interactions, and is associated with a variety of diseases including gastroenteritis and enteric fever. One of the first attempts to profile genome-wide expression changes in host cells during interaction of pro- and eukaryotes was carried out with *S. enterica* and human intestinal epithelial cells [2]. Conversely, the transcriptome of *S. enterica* became subject to several studies of host-pathogen interactions after completion of the genome sequences of *S. enterica* serotype Typhi CT18 [3] and serotype Typhimurium LT2 [4], which complemented some of the previously determined host responses [5,6]. In the meantime, next-generation sequencing (NGS)-coupled transcription profiling techniques emerged as the principal tools to interrogate gene expression, and especially whole transcriptome shotgun sequencing (RNA-Seq) has considerably contributed to our understanding of prokaryotic transcriptomes [7,8]. Nonetheless, simultaneous transcription profiling without prior disruption of the interaction remains technically challenging, and thus characterization of disease-related expression patterns in interacting eu- and prokaryotic cells is inevitably linked to comprehensive sequencing efforts [9].

Here we present dual 3’Seq, a tag-based, NGS-coupled method that allows for simultaneous transcription profiling of interacting pro- and eukaryotes without physical separation of the interacting cells. Compared to RNA-Seq, the reduction in complexity of tag-based approaches significantly decreases the required sequencing depth for a good coverage of both the pro- and eukaryotic transcriptomes [10-12], which is a prerequisite for profiling of low abundant pathogen-derived transcripts. Additionally, only a single tag is generated out of each transcript, which facilitates unequivocal quantification of reads from a specific RNA without sacrificing qualitative information of pathogen-derived transcripts, since prokaryotes lack alternative splicing events [13]. DeepSuperSAGE (Serial Analysis of Gene Expression; see [14-16]) and MACE (Massive Analysis of cDNA ends; see [17]) represent two established NGS-coupled transcriptome profiling techniques that generate exactly one tag out of the 3′ end of every transcript. While deepSuperSAGE yields a 26 nucleotide tag that is specifically located within the 3′ end depending on the presence of an according restriction site for the employed anchoring enzyme, MACE generates randomly distributed tags out of the last hundreds of bases. MACE consequently allows for preparation of libraries with varying read lengths and provides additional information regarding transcripts that do not possess an according restriction site for anchoring. In order to assess the respective efficiencies in transcriptome profiling of cultivated and interacting *S. enterica* Typhimurium and human host cells, we combined the dual 3’Seq approach with both protocols. Human epithelial cells (HeLa-S3) were infected with the invasive pathogen *S. enterica* Typhimurium SL1344 (henceforth termed SL1344), and interacting cells were screened for differentially expressed transcripts at several points of time post infection to provide an overview of the transcriptional processes during invasion of the intestinal epithelium as one of the first steps in emerging salmonellosis. The combination of the published SL1344 transcriptome [18] with the operon structure identified by differential RNA-seq (dRNA-Seq; see [19]) allowed for accurate quantification of polycistronically transcribed genes from the prokaryote, and the corresponding expression profiles provide a basis for time-dependent analysis of disease-related transcripts, despite the fact that transcripts from the pathogen are of extremely low abundance during interaction.

## Results and discussion

### Dual 3’Seq of interacting pro- and eukaryotes

**Figure 1:**
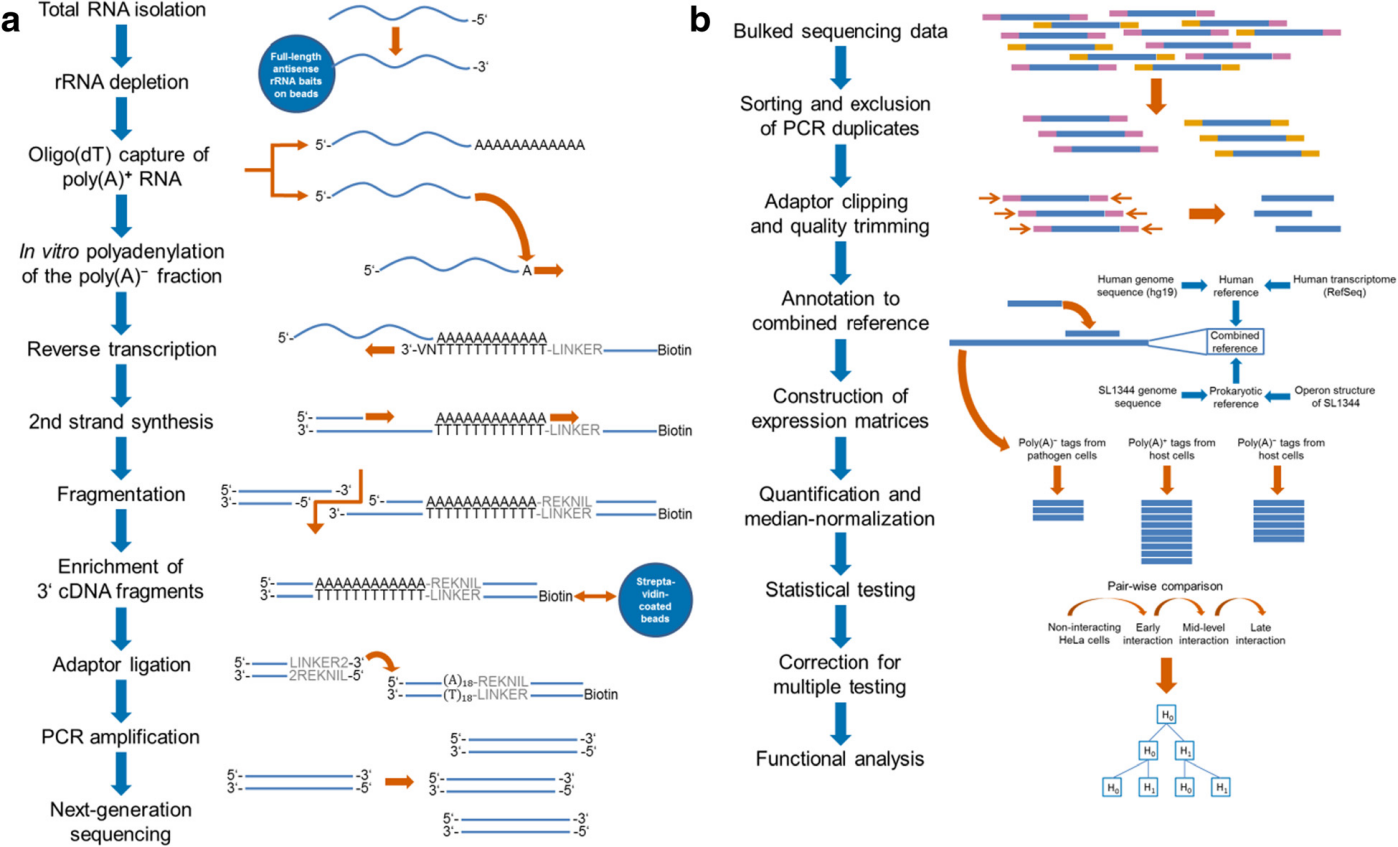
Scheme of dual 3’Seq library preparation and bioinformatic processing of the generated sequencing data. **(a)** Total RNA was size-selected (Additional file 3) subsequent to DNase I digestion of remaining DNA in the isolate. Following rRNA depletion (Additional file 3), the RNA was split into the poly(A)^+^ and poly(A)^−^ fraction by oligo(dT) capture to separate the polyadenylated and functional mRNAs of eukaryotic cells from the non-polyadenylated transcripts that represent the functional transcriptome of prokaryotes. Ensuing in-vitro polyadenylation of the poly(A)^−^ fraction, both fractions were subjected to oligo(dT)-based reverse transcription. The generated cDNA was fragmented according to two established 3′ transcriptome profiling techniques. DeepSuperSAGE tags were generated via cleavage of RNAs by the anchoring enzyme NlaIII and subsequent digestion using EcoP15I, while MACE involved random fragmentation for generation of tags. 3′ fragments were enriched by binding to a streptavidin matrix and ligated to a sequencing adaptor. Adaptor-ligated fragments were PCR-amplified using GenXPro’s TrueQuant technology for PCR-bias free amplification, PAGE-purified, and sequenced on the Illumina HiSeq2000 platform. **(b)** Barcoded reads were allocated to their respective library, filtered for PCR-derived reads, and trimmed for high-quality sequences. Afterwards, reads were annotated to a combined reference comprising the transcriptome and genome sequences of SL1344 and human host cells in a multi-step procedure. Reads uniquely mapped to one of both organisms were combined to three distinct expression matrices for functional analysis of the poly(A)^−^ transcriptome from pathogen and host cells as well as the poly(A)^+^ fraction of the host cells. For each expression matrix, annotated reads were quantified and median-normalized using DESeq, followed by pair-wise, time-dependent comparison of the different interaction stages. Statistical significance was subsequently corrected for multiple testing according to Benjamini and Hochberg.

In order to reduce the sequencing depth for simultaneous characterization of the transcriptomes of interacting pro- and eukaryotic cells without prior separation, we established the new transcriptome profiling technique dual 3’Seq (Figure 1). We employed this technique to identify time-dependent transcription patterns of interacting HeLa-S3 and SL1344 cells subsequent to infection of the host cells. Total RNA from cultured and interacting cells was isolated after different points of time to interrogate the molecular events during early, mid-level and late interaction stages of infection (0.5, 4, and 24 hours post infection, respectively). The first interaction point was chosen as early as technically possible to capture the initial pathogen responses after invasion of the host cells, while mid-level and late interaction allow for investigation of the early and late host cell response as well as intracellular pathogen replication.

**Figure 2:**
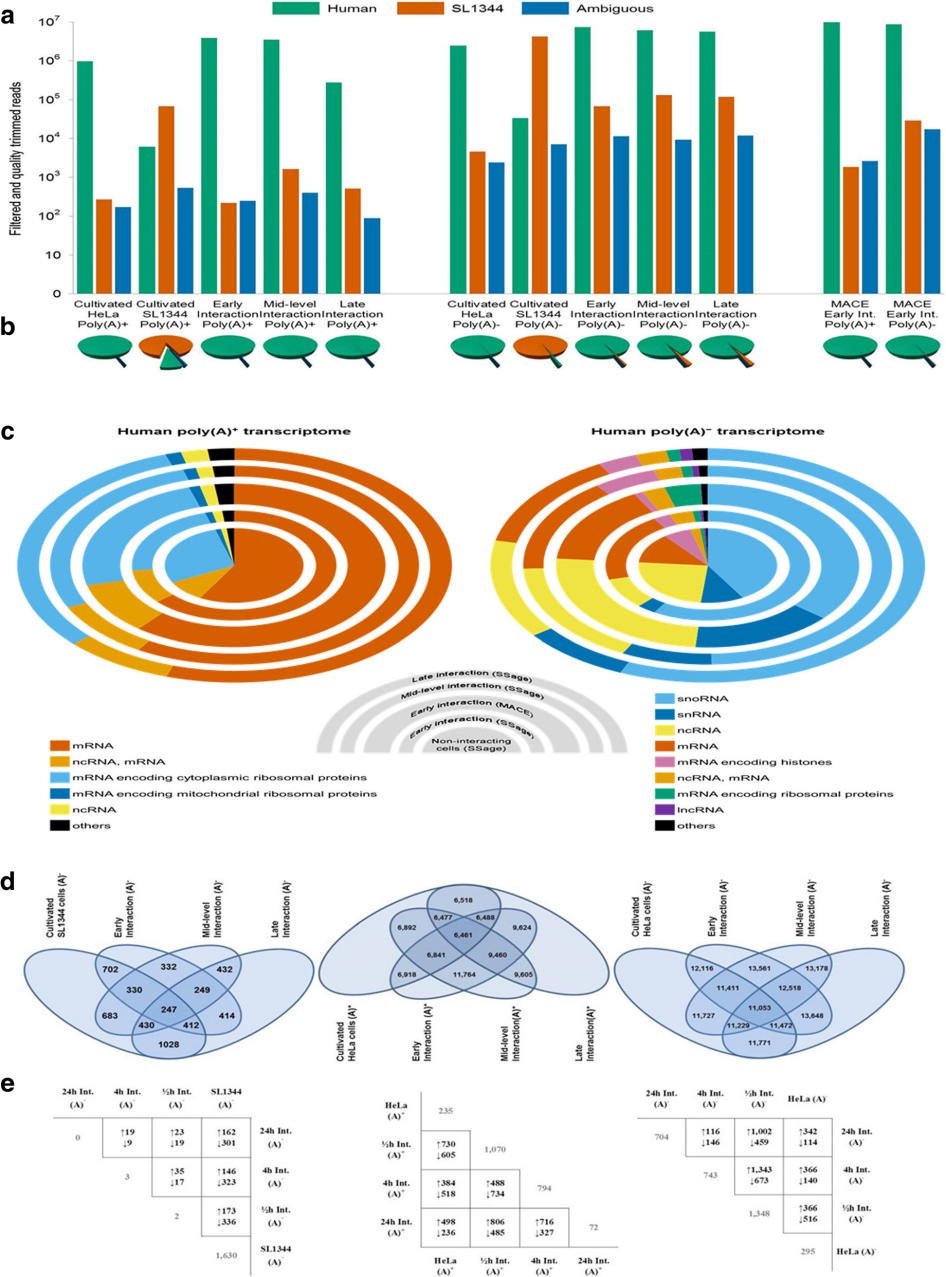
Annotation statistics for the generated expression matrices and differentially expressed genes of interacting pathogen and host cells. **(a**,**b)** Read numbers annotated to human (green) and pathogen (brown) cells or both (blue) for all sequenced libraries, respectively. The bar graph shows log_10_-transformed numbers, while the original number of reads is depicted in a circle graph. **(c)** Classification of unambiguously annotated transcripts from host cells into mRNAs (vermillion), non-coding RNAs (yellow), bivalent transcripts encoding mRNAs and non-coding RNAs (orange), long non-coding RNAs (purple), histone-encoding mRNAs (reddish purple), and other transcript classes (black) for the poly(A)^+^ and poly(A)^−^ transcriptome, respectively. mRNAs encoding cytoplasmic (skyblue) or mitochondrial (blue) ribosomal proteins are depicted individually for the poly(A)^+^ and combined (bluish green) for the poly(A)^−^ transcriptome that additionally comprises small nucleolar RNAs (skyblue) and small nuclear RNAs (blue). Respective abundances are shown for non-interacting (circle graphs) as well as interacting (radial graphs) cells. The different interaction stages (early, mid-level, and late interaction) prepared with 3’Seq coupled to deepSuperSAGE (SSage) are depicted from inside outwards along with the early interaction library that was prepared using MACE (doubled width). **(d)** Venn diagram showing the number of commonly expressed genes for all combinations of libraries corresponding to one of the expression matrices. Ambiguously annotated reads are not included. **(e)** Pair-wise comparison of differentially expressed transcripts within the expression matrices (black) along with the number of exclusively expressed transcripts (gray) for each library. Listed are unambiguously annotated reads with a log_2_ fold change stronger than |1.5| and an FDR-corrected p-value below 0.05. Up- and downregulated transcripts are indicated by corresponding arrows for the library on the ordinate, respectively.

The generated sequencing data from the poly(A)^−^ fractions comprise between 2.5 and 7.5 million high-quality and PCR duplicate-free reads, while the number of reads in the corresponding poly(A)^+^ libraries ranges from almost 4 million down to ~75,000 reads in the SL1344 library (Figure 2a,b and Additional file 1: Table S1). The relatively low number of filtered poly(A)^+^ reads from cultivated and especially interacting SL1344 cells reflects post-transcriptional regulation in prokaryotes, where polyadenylation facilitates degradation of RNA [20]. Additionally, the low abundance of polyadenylated intermediates from the prokaryote is corroborated by the low ratios of SL1344-derived transcripts in all interaction stages (<0.2% of all reads), which impedes a functional analysis of this fraction. Prokaryotic reads in libraries of the poly(A)^−^ fraction are about ten times more abundant (~1-2%). Compared to the ratios of the corresponding poly(A)^+^ libraries, non-polyadenylated SL1344 reads are about 160-fold, 50-fold and 10-fold higher from early to late interaction, respectively. This increase in polyadenylated reads during interaction suggests an increased turnover of transcripts probably caused by the growing number of (stressed) pathogen cells in the confined environment of HeLa cell culture. Discarding ambiguously annotated poly(A)^−^ reads that aligned to more than one gene, 4,555 or 89% of the 5,137 known loci in SL1344 are transcribed in cultivated or interacting cells, while 14,343 or 75% of 19,233 protein-coding loci are represented in the polyadenylated transcriptomes of human host cells. Over 95% of the transcripts in human poly(A)^+^ libraries encode proteins, and mRNAs encoding ribosomal proteins account for a large proportion of these (Figure 2c). With an mRNA content ranging from 13% to 20% protein-coding transcripts are much less abundant in the corresponding poly(A)^−^ libraries, which mostly comprise small nucleolar RNAs (snoRNA), small nuclear RNAs (snRNA) and other non-coding RNAs (ncRNA).

### Differential gene expression during interaction

The intersections of unambiguously annotated RNAs from the poly(A)^−^ transcriptome of SL1344 cells as well as both transcriptome fractions of human host cells reveal distinct overlaps of the expressed genes (Figure 2d). The four SL1344 libraries comprise ~250 commonly expressed genes, while non-interacting and late interacting cells share the highest number with more than 1,000. Unsurprisingly, cells from early and mid-level interaction express fewer genes in common with non-interacting cells than those from the late interaction stage when infection is well established. Conversely, the number of commonly expressed genes in the poly(A)^+^ transcriptome of host cells is highest between early and mid-level interaction (approximately 12,000 versus 6,500 common genes in all four libraries). With 12,350 ± 1,300 genes, the numbers of overlapping genes in the poly(A)^−^ fraction of host cells spread notably less than in the poly(A)^+^ fraction, which suggests a more diverse effect of infection on the polyadenylated transcriptome of host cells. Although the poly(A)^−^ fraction of host cells generally comprises more significantly up and downregulated transcripts than the poly(A)^+^ fraction (Figure 2e), expression of polyadenylated transcripts during infection is more affected, since mRNA degradation naturally leads to corresponding changes in the non-polyadenylated transcriptome. In fact, more than 80% of the non-polyadenylated, significantly differentially expressed transcripts in all the pairwise comparisons represent protein-coding mRNAs, even though these RNAs only constitute up to a fifth of their respective poly(A)^−^ fractions in total (Figure 2c).

**Figure 3:**
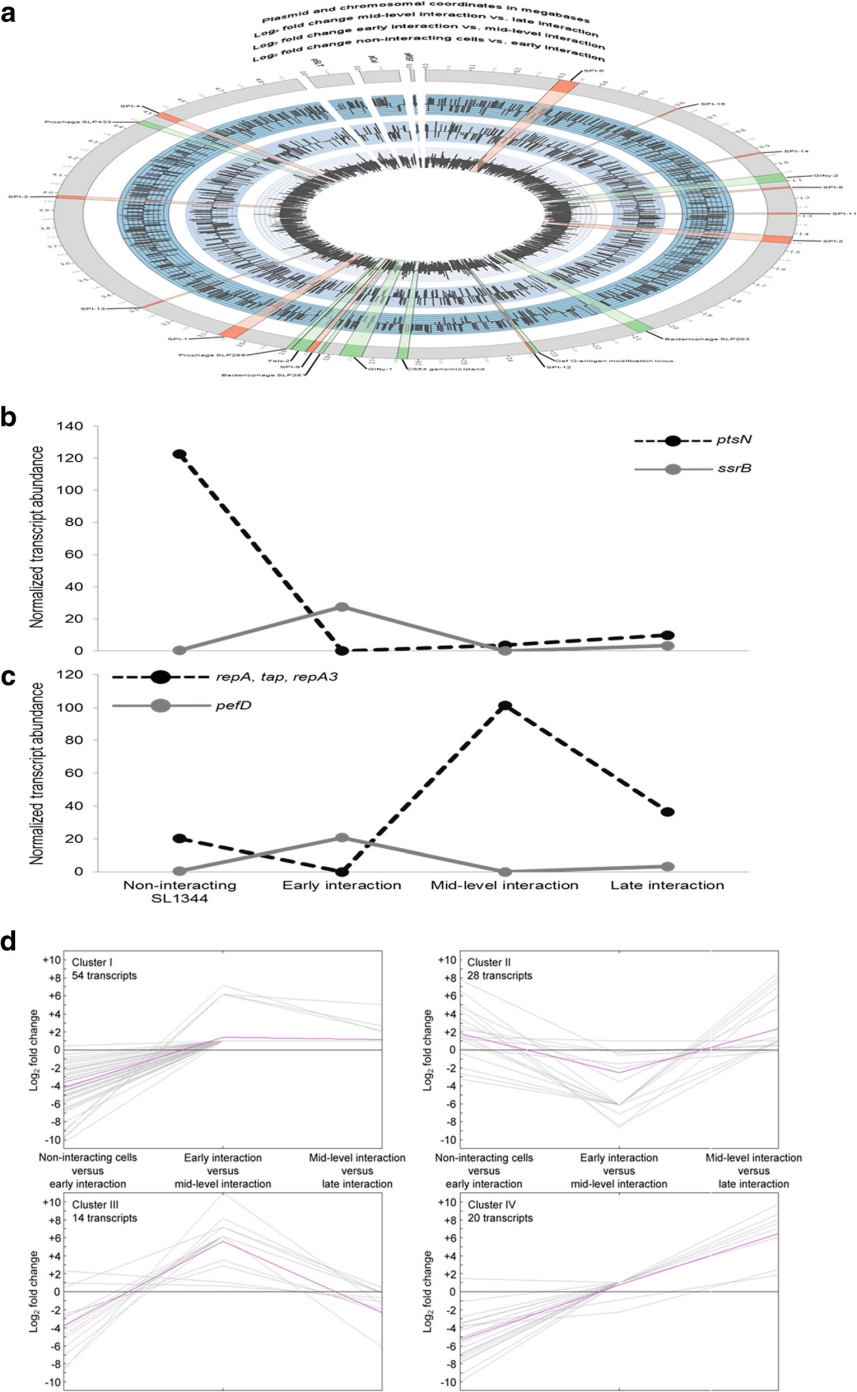
Differential expression of interacting pathogen cells. **(a)** Circos plot of SL1344 gene expression for chromosomally-encoded genes as well as plasmid-derived transcripts. The respective coordinates are given in megabases (outmost gray circle) along with the corresponding log_2_ fold changes of non-interacting and early interacting, early and mid-level interacting, as well as mid-level and late interacting cells (inner to outer blue circles, respectively). *Salmonella* pathogenicity islands (SPIs) are highlighted in orange, while other important loci are shown in green. **(b**,**c)** Detailed time-dependent expression profiles of selected SL1344 genes. The median-normalized transcript abundance is shown for non-interacting cells along with those from the different interaction stages. *PtsN* is transcribed as polycistronic mRNA from the chromosome that comprises ten other genes (*yrbG*, *yrbH*, *yrbI*, *yrbK*, *yhbN*, *yhbG*, *rpoN*, *yhbH*, *yhbJ*, and *ptsO*). The mRNA encoding pefD and the polycistronic mRNA coding for repA, tap, and repA3 are both transcribed from intracellular plasmid pSLT^SL1344^. **(d)** K-means clustering of the time-dependent fold changes from plasmid-encoded transcripts. The corresponding log_2_ fold changes were assigned to one of four clusters based on their time-dependent expression patterns. The closest centroid (purple) is shown along with the assigned transcripts (gray) for each cluster. Clustering was performed with Pearson Correlation as distance metric. An accordingly sorted list of the clustered transcripts is provided in Additional file 2: Table S2.

Expression of *STM2239* and *STM1015* is non-detectable except for the transcriptome of early interacting SL1344 cells, while the transcripts encoded by *gst*, *ydiN* and *STM1029* are only present in mid-level interaction (Figure 2e). *STM2239* is part of *Salmonella* pathogenicity island 12 (SPI-12), and substantially contributes to bacterial fitness by promoting transcription of SPI-12 together with *ssrB* [21]. *STM1015* and *STM1029* are both encoded within Gifsy-2, a lambdoid prophage that promotes virulence in addition to the remnant phage encoded by SPI-12 [22]. The time-dependent differential expression of all sequenced genes from the prokaryote reveals characteristic patterns for each interaction stage (Figure 3a). The expression of many SPI-encoded and phage-derived transcripts from the chromosome as well as plasmid-derived transcripts is induced or repressed in the course of infection, and analysis of these disease-related expression patterns allows for dissolving the time-dependent components of SL1344 gene expression during different stages of infection.

### Time-dependent expression patterns of the prokaryotic pathogen

Expression of virulence genes from the pathogen is tightly regulated, depending on the respective environment, and the SsrB response regulator of the SsrA/SsrB two-component system represents one of the major virulence modulators that controls about 4% of the *S. enterica* genome including *ssrB* itself and genes in various SPIs [23]. The first two SL1344 pathogenicity islands (SPI-1 and SPI-2) encode two distinct classical type III secretion systems [24]. These systems represent needle-like structures that inject effector proteins into the cytoplasm of host cells, which are necessary for invasion and subsequent proliferation of the pathogen. Expression of SPI-2 genes is controlled by the SsrA/SsrB along with the OmpR/EnvZ and PhoP/PhoQ two-component systems [25]. The SsrB response regulator, however, exhibits the most direct effect on SPI-2 expression, and is negatively controlled by *ptsN*-encoded EIIA^Ntr^, a component of the nitrogen-metabolic phosphotransferase system that acts on the SsrB protein at the post-transcriptional level [26]. In line with this, *ptsN* and *ssrB* display a reciprocal time-dependent expression (Figure 3b). The *ptsN* mRNA is most abundant in non-interacting SL1344 cells, absent during early interaction, and slightly upregulated in the subsequent course of infection. Conversely, the transcript encoding SsrB is most abundant during early interaction, but not detectable in non-interacting cells and mid-level interaction.

Fold changes of transcripts expressed from one of the three plasmids (pSLT^SL1344^, pCol1B9^SL1344^, and pRSF1010^SL1344^) of non-interacting and early interacting, early and mid-level interacting, as well as mid-level and late interacting SL1344 cells were subjected to *k*-means clustering (*k* = 4) to identify transcripts with similar time-dependent expression patterns (Figure 3d, Additional file 2: Table S2). *Salmonella* virulence plasmids are very stable and present in low copy numbers (1–2 per chromosome; see [27]). The generated clusters consequently reflect the temporal contribution of plasmid-encoded genes to the virulence program of SL1344. Transcripts relevant for invasion of host cells and early adaption of endocytosed SL1344, for instance, are represented in cluster II. Upregulation of some of these transcripts from mid-level to late interaction additionally suggests a function of the encoded proteins in later stages of infection, and amongst others this cluster includes the mRNA for plasmid-encoded fimbriae D (PefD). Cluster III, on the other hand, comprises transcripts that are especially involved in mid-level interaction. In this cluster, the most upregulated transcript from early to mid-level interaction represents a polycistronic mRNA encoding the proteins RepA, Tap, and RepA3 that are required for plasmid replication. Transcripts less involved in early or mid-level interaction, but becoming successively more important with proceeding invasion and infection of host cells are represented in cluster IV. Amongst others, this cluster includes the mRNA encoding plasmid SOS inhibition protein A (PsiA), and the most downregulated and subsequently upregulated mRNA, which encodes a putative transposase (PSL035). With 54 out of 116, cluster I comprises almost half of the identified plasmid-encoded transcripts. However, cluster I also displays the least consistent gene expression pattern. Only four of the 54 transcripts are also found to be expressed in interacting cells indicating that those transcripts, which could not reliably be quantified due to insufficient sequencing depth, were grouped into this cluster. Figure 3c displays the time-dependent expression profiles of the mRNAs encoding RepA, Tap, and RepA3 as well as PefD in more detail. The fimbrial chaperone protein PefD is involved in the F1–G1 short (FGS)-assisted assembly of thick rigid mono-adhesive pili, which represent adhesive organelles necessary for bacterial attachment to target cells [28]. In line with this, *pefD* expression is not detectable in non-interacting cells, highly induced in early interaction, and non-detectable during mid-level interaction again. The transcript encoding RepA, Tap, and RepA3, on the other hand, is expressed in non-interacting cells, completely absent during early interaction, highly abundant in the course of intracellular replication during mid-level interaction, and finally less abundant in late interaction again.

The identified expression patterns of interacting pathogen cells reflect previous reports of infection-related gene expression and function, which corroborates the potential of dual 3’Seq to reliably assess the transcriptome of prokaryotic cells during interaction, including quantification of polycistronic transcripts.

### Host cell responses to bacterial infection

Innate immune recognition of microbial components relies on germline-encoded pattern-recognition receptors (PRRs) that recognize pathogen-associated molecular patterns (PAMPs) of foreign cells such as bacterial lipids, lipoproteins, proteins, nucleic acids and lipopolysaccharides (LPSs), the constituents of the outer membrane of Gram-negative bacteria [29]. Toll-like receptors (TLRs) represent the most important family of membrane-bound PRRs, and among the ten functional TLRs in humans TLR4 is unique in its ability to induce two distinct signaling pathways controlled by the TIRAP-MyD88 and TRAM-TRIF pairs of adaptor proteins [30]. Binding of bacterial LPS to MD2 and TLR4 induces TIRAP-MyD88-dependent signaling at the plasma membrane, and subsequently activates the TRAM-TRIF pathway after endocytosis of TLR4. In HeLa cells, however, the genes encoding MD2 as well as TLR2 are not transcribed [31] and consequently immune recognition of the pathogen must involve other PRRs.

**Figure 4:**
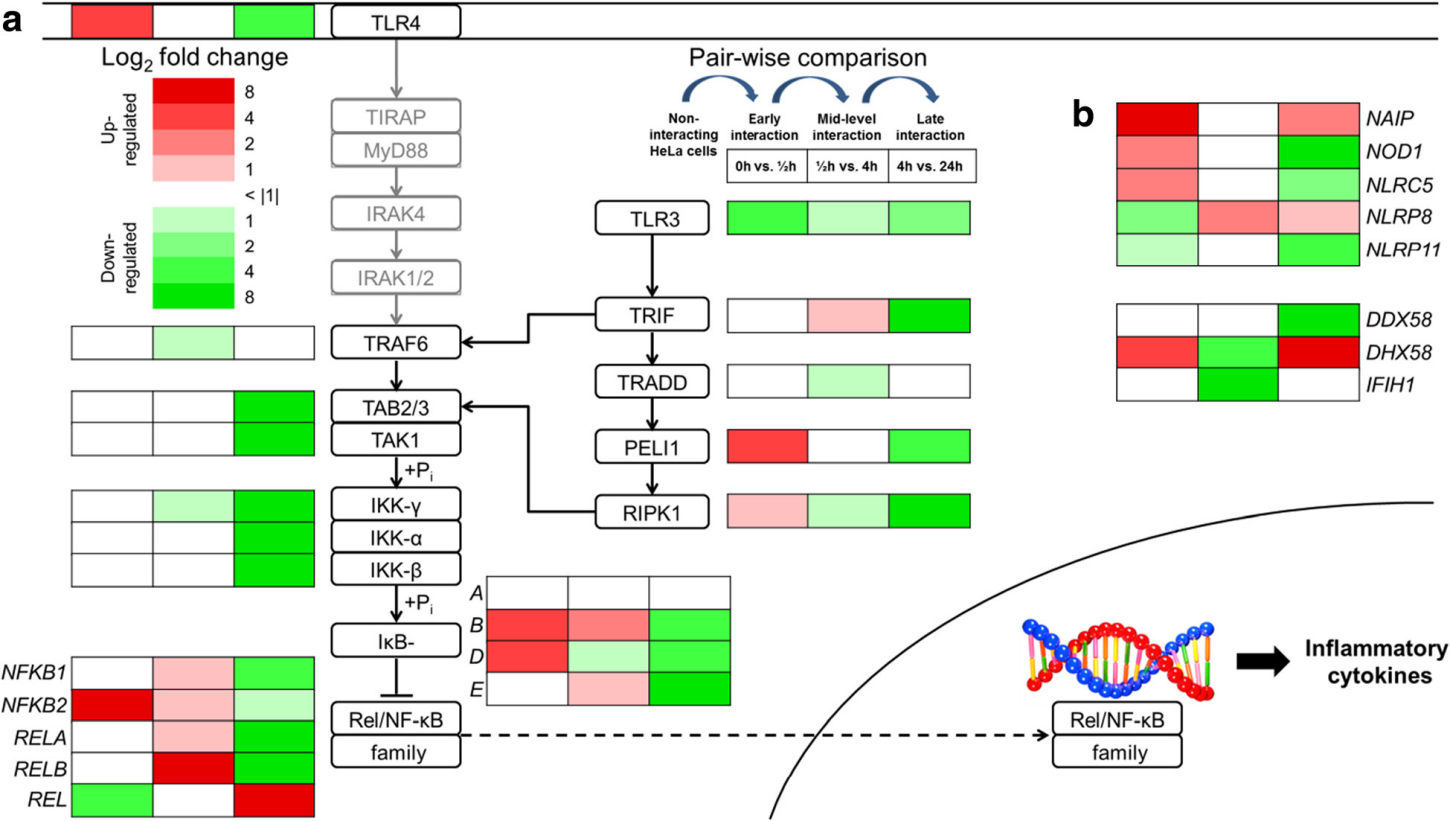
Differential expression of membrane-bound and cytosolic PRRs in response to infection. Time-dependent log_2_ fold changes of mRNAs from non-interacting and interacting cells are depicted for the listed gene products, and interactions between the encoded proteins are indicated by arrows. **(a)** Differentially expressed mRNAs encoding proteins involved in early (MyD88-dependent) and late (TRIF-dependent) NF-κB signaling. Binding of bacterial LPS to TLR4 activates two distinct signaling cascades that lead to expression of inflammatory cytokines via activation of the Rel/NF-κB transcription factor family. Activation of MyD88 results in signal transmission via interleukin-1 receptor-associated kinases (IRAKs) that in turn activate TRAF6. This part of the signaling pathway, however, is not functional in HeLa cells (gray boxes) because of lacking *MD2* expression. TRAF6 signaling results in activation of the kinase TAK1 complex that phosphorylates NEMO (IKK-γ), which in turn leads to phosphorylation of the inhibitor subunits of NF-κB (IκBs) via canonical IKK-β. Phosphorylated IκBs are degraded by the ubiquitin-dependent pathway, thereby releasing NF-κB, which subsequently translocates to the nucleus to function as a transcription factor (dashed arrow). TRIF-dependent signaling is also stimulated via activation of TLR3 by binding of double stranded RNA, and enhances activation of the kinase TAK1 complex by formation of a multimeric protein signaling complex comprising TRAF6, TRADD, Pellino-1 (*PELI1*) and RIP1 (*RIPK1*). **(b)** Differential expression of other cytosolic PRRs that modulate the NF-κB response by cross-signaling. Nod-like receptors comprise four subfamilies, and expression profiles of the mRNAs encoding NAIP (NLRB family), NOD1, and NLRC5 (NLRC family) as well as NLRP8 and NLRP11 (NLRP family) are shown along with those from the RIG-I-like receptors LGP2 (*DHX58*), RIG-I (*DDX58*), and MDA5 (*IFIH1*).

The mRNAs encoding several members of the Rel/NF-κB transcription factor and NF-κB inhibitor family display a marked response in gene expression upon infection (Figure 4a). Especially, the differential expression patterns of mRNAs encoding inhibitor proteins (IκBs) point to an increase in IκBs marked for degradation subsequent to activation of the pathway with the onset of infection. Except for a general trend to downregulation from mid-level to late interaction, expression levels of transcripts coding for other members of the TLR4 signaling cascade are largely unaffected in the different interaction stages, which is in line with the fact that most of the encoded proteins are not degraded or depleted from a cytosolic pool in the context of signal transmission. Besides TLR3 and TLR7-9 that are exclusively expressed in intracellular vesicles, host cells harbor additional classes of cytosolic PRRs, including RIG-I-like receptors (RLRs) and Nod-like receptors (NLRs) [29]. In contrast to TLRs, RLRs are present in the cytosol of all cell types, and activation by viral, but also bacterial nucleic acids results in induced expression of type I interferon and cytokines [32]. The transcript encoding LGP2 (*DHX58*) is strongly upregulated in the first and last infection stage, while its expression is downregulated from early to mid-level interaction (Figure 4b). The differential time-dependent expression of this transcript, in contrast to other RLR-encoding mRNAs as MDA5 (*IFIH1*) and RIG-I (*DDX58*), suggests a pathogen-induced activation of LGP2 and points to a functional role of the protein in early and late responses to infection. The NLR family comprises more than 20 members for recognition of various PAMPs, and the identified expression profiles of corresponding mRNAs differ accordingly. The transcript encoding NAIP displays the strongest upregulation from non-interacting to early interacting cells, and is additionally upregulated from mid-level to late interaction. Time-dependent expression of this mRNA consequently resembles the pattern of *DHX58*, again suggesting a functional implication of the encoded protein in response to infection.

Distinct library preparation of the poly(A)^+^ and poly(A)^−^ fraction by dual 3’Seq allows for discriminating polyadenylated (pri-miRNA) from non-polyadenylated (pre-miRNA) microRNA (miRNA) precursors. A previous study of the SL1344-induced microRNA response of HeLa cells identified the let-7 family as an important regulator of major cytokines [33]. In line with this, the host gene encoding miRNA let-7 g (*MIRLET7BHG*) is expressed strongest in non-interacting cells and continuously repressed throughout the interaction stages (data not shown). While the level of let-7 g pre-miRNA is relatively stable during interaction, the pri-miRNA abundance is halved with each point of time post infection. MiR-210 is involved in the response of human cells to infection with *Leishmania major* [34], and the fact that the pri-miRNA encoded by *MIR210HG* is most abundant during late interaction of human host and SL1344 cells (3.3-fold upregulated versus mid-level and 2-fold upregulated versus early interaction in log_2_ scale) suggests an additional role of this miRNA in the context of bacterial infection. Taken together, deepSuperSAGE-coupled dual 3’Seq provides insights into host cell responses to bacterial infection with minimal sequencing efforts not only for the protein-coding transcriptome, but also for ncRNAs such as miRNA precursors.

### Validation of time-dependent expression patterns and assessment of the quantification accuracy of dual 3’Seq by quantitative real-time PCR

The identified time-dependent expression patterns from pathogen and host cells were confirmed by MIQE-conform qRT-PCR [35] with three independent biological replicates. *GAPD*, *B2M* and *RPL13A* served as reference genes for normalization of the transcript abundances in host cells. Out of seven previously determined candidate genes for normalization of prokaryotic transcript abundances *nagD*, *ndh*, *rpoD*, and t*rmA* exhibited the highest expression stabilities between pools of reverse-transcribed cDNA from the three biological replicates of each interaction stage as well as non-interacting cells and between the three distinct biological replicates of non-interacting cells (Table 1).

**Table 1:** **Relative expression stabilities of seven candidate reference genes from prokaryotic cells according to Bestkeeper, NormFinder, and GeNorm**

**Evaluation method**	Candidate reference gene stability*
Bestkeeper	***trmA*** *,* ***nagD*** *,* ***ndh*** *,* ***rpoD*** *, ygfE, rpsO, rpsT*
NormFinder	***rpoD*** *,* ***ndh*** *,* ***nagD*** *,* ***trmA*** *, ygfE, rpsO, rpsT*
GeNorm	***trmA*** */* ***nagD*** *,* ***ndh*** *,* ***rpoD*** *, ygfE, rpsO, rpsT*

*Gene stability decreasing from left to right; the employed reference genes for quantification of the time-dependent expression from prokaryotic transcripts are indicated by bold letters.

Please consult the text for more information.

**Figure 5:**
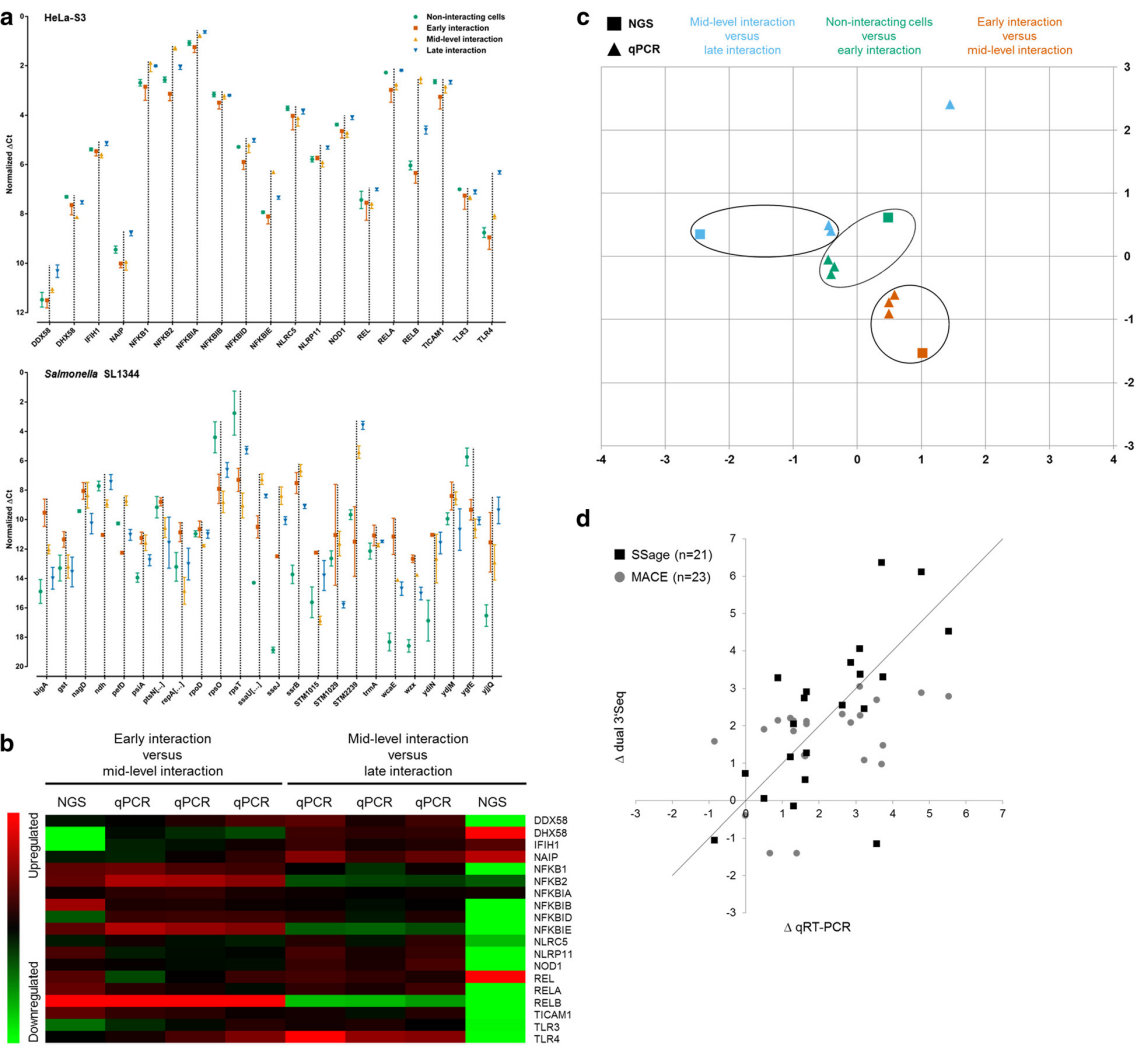
Confirmation of time-dependent expression patterns from pathogen as well as host cells and evaluation of dual 3’Seq quantification accuracy by quantitative real-time PCR. **(a)** Biological variance of the targeted transcripts across three independent biological replicates. Means with SD of the ∆Ct values from non-interacting cells (green) and cells from early (vermillion), mid-level (orange) and late interaction (blue) are shown for 19 mRNAs involved in host cell responsaes to bacterial infection (upper graph) as well as 24 prokaryotic transcripts (lower graph). Additional coding sequences of polycistronic transcripts from the prokaryote are indicated by brackets. ∆Ct values are plotted on an inverted ordinate, where a low figure reflects an increased abundance of the respective transcript and vice versa. For each graph, the lowest Ct value was arbitrarily shifted to zero followed by an according normalization of the other values. **(b)** Heat map of time-dependent log_2_ fold changes and corresponding ∆∆Ct values from early and mid-level interacting as well as mid-level and late interacting host cells. The individual expression ratios determined by qRT-PCR from three biological replicates (qPCR) together with the corresponding ratios identified by deepSuperSAGE (NGS) are depicted after hierarchical clustering of all samples based on Uncentered Pearson Correlation. Upregulated transcripts are represented in red, while downregulated transcripts are shown in green. **(c)** Principal component analysis of the data included in the heat map along with the corresponding log_2_ fold changes from non-interacting and early interacting cells (green). **(d)** Scatterplot depicting the relative quantification accuracy of dual 3’Seq in comparison to probe-based qRT-PCR. The expression ratios of 23 polyadenylated mRNAs and their non-polyadenylated degradation intermediates in early interacting cells determined either by deepSuperSAGE (SSage) or MACE (y-axis) is plotted against the respective qRT-PCR-based ratios (x-axis). An optimal correlation is indicated by the diagonal line.

The biological variance of 19 target mRNAs involved in host cell responses to bacterial infection is relatively small compared to the variance of 20 target transcripts from the pathogen (Figure 5a). An exact qRT-PCR-based quantification of prokaryotic transcripts is apparently impeded by the high Ct values that are associated with the relatively low ratio of pathogen-derived transcripts in all interaction stages. Clustering of the time-dependent differential expression from the 19 human mRNAs either determined by dual 3’Seq coupled to deepSuperSAGE or by qRT-PCR, on the other hand, reveals a good correlation especially for the mRNAs encoded by *NFKB1*, *NFKB2*, *NFKBIE* and *RELB* (Figure 5b). According to this, the initial immune response of host cells involves activation of p50 (*NFKB1*) and p52 (*NFKB2*) that act as transcriptional activators or repressors depending on the respective dimerization partner, along with I-Rel (*RELB*), which exhibits the most prominent differential expression of all 19 mRNAs. Since a consistent differential expression of these mRNAs across all three biological replicates is only present from early to mid-level and from mid-level to late interaction, the initial host cell response to SL1344 infection seems to occur in between half an hour and four hours post infection. Principal component analysis of the 19 human mRNAs demonstrates that the time-dependent differential expression measured by qRT-PCR of the biological replicates is similar to the respective expression patterns determined by dual 3’Seq (except for one outlier regarding mid-level versus late interaction), and allows for discriminating the different interaction stages from each other (Figure 5c).

Quantification accuracy of dual 3’Seq coupled either to deepSuperSAGE or alternatively to MACE was assessed by probe-based qRT-PCR of 23 mRNAs with DNase-treated and rRNA-depleted total RNA from library preparation of the early interaction stage. The ratios between functional mRNAs and their non-polyadenylated degradation intermediates were determined in one-step qRT-PCR reactions and plotted against the identified ratios according to deepSuperSAGE or MACE, respectively (Figure 5d). Even though two of the targets (*APOD* and *ITGB3*) are not present in the sequencing data of deepSuperSAGE-coupled library preparation, the Pearson correlation is better than the correlation of MACE-based dual 3’Seq (0.68 versus 0.45, respectively). Four out of the 21 targets captured by deepSuperSAGE exhibit an almost optimal correlation with qRT-PCR, and especially the transcript encoded by *SLC7A11* is found to be more abundant in the non-polyadenylated transcriptome by both methods.

## Conclusions

The identified ratios between human transcripts and transcripts originating from SL1344 in interacting cells demand extensive sequencing to sufficiently cover the transcriptome of interacting pathogen cells without their prior enrichment. Compared to the eukaryote, reads from SL1344 exhibit a maximal ratio of 1 to 50. Additionally, SL1344-derived reads in the poly(A)^+^ fraction are notably less abundant (between 0.01% and 0.18% of all reads) compared to the corresponding poly(A)^−^ fraction (with 0.9% up to 2.1%). The latter fraction is crucial for functional analysis of differentially expressed transcripts that encode proteins involved in infection, since polyadenylated transcripts from the prokaryote merely represent degradation intermediates. Distinct library preparation with the poly(A)^+^ and poly(A)^−^ fraction of interacting cells via dual 3’Seq preserves the relatively high percentages of SL1344-derived transcripts in the non-polyadenylated fraction of host cells, and consequently permits a more detailed analysis of the functional, non-polyadenylated prokaryotic transcriptome during interaction. In contrast, combined library preparation of both fractions inevitably leads to a reduced ratio of pathogen transcripts due to the immense diversity of the polyadenylated host cell transcriptome on the one, and the extremely small number of polyadenylated intermediates from the pathogen, on the other hand.

Reciprocal expression of *ptsN* and *ssrB* proves that polycistronic transcripts from the prokaryote were accurately quantified, since *ptsN* is transcribed as polycistronic mRNA that encodes ten other gene products, while *ssrB* is expressed as a monocistronic transcript. The efficiency of in-silico separation of the two interacting organisms can be assessed through the number of false positive annotated reads in the libraries of non-interacting cells (Figure 2b, Additional file 1: Table S1). Although, the poly(A)^+^ fraction of non-interacting SL1344 cells contains a relatively high percentage of incorrectly aligned reads (8.3% mapped to the human reference), the corresponding poly(A)^−^ fraction only comprises 0.8% of false positive reads. With 0.03% in the poly(A)^+^ and 0.18% in the poly(A)^−^ fraction, libraries from non-interacting host cells comprise even less incorrectly aligned reads. Thus, the high ratio of false positive reads in the poly(A)^+^ fraction of non-interacting SL1344 cells is not representative for the general efficiency of the employed annotation procedure, but rather caused by the extremely low coverage of this library and sequencing of degraded intermediates.

Mapping of 3’ reads with a length of more than 26 nucleotides did not substantially improve the ratio of transcripts that aligned to both the human and the SL1344 transcriptome as can be inferred from the numbers of reads excluded during multi-step annotation (Figure 2a, Additional file 1: Table S1). The corresponding percentages of excluded reads from early interaction libraries prepared either with deepSuperSAGE or MACE are relatively similar (0.01% vs. 0.03% in the poly(A)^+^ and 0.15% vs. 0.20% in the poly(A)^−^ fraction, respectively). However, generation of tags via deepSuperSAGE depends on the presence of a recognition site for the anchoring enzyme, while MACE generates a tag out of every transcript. The comparison of both methods with qRT-PCR confirms that not all expressed transcripts are represented in the deepSuperSAGE dataset, but also that the captured transcripts are quantified accurately by deepSuperSAGE-coupled dual 3’Seq. Transcriptome profiling by deepSuperSAGE consequently provides a reliable representation of the differential expression from interacting pro- and eukaryotic cells, but it also involves a much more elaborate library preparation procedure compared to MACE. Another advantage of the latter technique is its potential to capture the expression of small ncRNAs, since it does not necessarily involve size-selection (see Additional file 3). Additionally, MACE can be adjusted to any desired sequencing length, which allows for identification of alternative polyadenylation sites in eukaryotes [36].

For functional analysis, the trade-off between sequencing depth and the number of biological replicates for each condition must be carefully balanced [37]. Consequently, every decrease in the necessary amount of sequencing per sample allows for a more detailed time-dependent analysis and more biological replicates per condition. Dual 3’Seq employing deepSuperSAGE significantly reduces the complexity of the involved transcriptomes. Even though the prokaryotic transcriptome of interacting cells was not completely covered, our approach provides first insights into the pathogenicity-related gene expression of SL1344 and corresponding host cell responses. The time-dependent expression patterns of SPI and plasmid-encoded transcripts in interacting SL1344 cells reflect a coordinated activation of virulence genes in the course of infection, which in turn elicits corresponding host responses in transcription as exemplarily shown for the signaling cascades leading to expression of inflammatory cytokines.

## Methods

### Human cell line and *S. enterica* strain

Infection assays were carried out with a HeLa-S3 cell line from LGC standards (ATCC CCL-2.2) to meet the specifications of the ENCODE project, and the *Salmonella enterica* subspecies I serotype Typhimurium strain SL1344 with a chromosomally integrated P_tet_::gfp construct (JVS-3858; see [38]).

### Microbiological methods and cell culturing

Culture of HeLa-S3 cells was routinely performed in T-75 flasks (Corning Inc.) with Dulbecco’s modified Eagle’s medium (DMEM; Gibco) supplemented with 10% fetal calf serum (Biochrom), 2 mM L-glutamine (Gibco) and 1 mM sodium pyruvate (Gibco) at 37°C in a humidified 5% CO_2_ atmosphere. For infection assays, cells were seeded in 6-well plates (Corning Inc.) in a density of 2×10^5^/well, 2 days prior to infection.

*S. enterica* Typhimurium SL1344 from a glycerol stock stored at −80°C was streaked onto Lennox broth (LB) plates, two days prior to infection of the human host cells. The plates were incubated overnight at 37°C. The next day, a single colony was used to inoculate an overnight culture in 3 ml LB medium. For preparation of the bacterial inoculum, SL1344 cells were resuspended in host culture medium (DMEM). Likewise, extracellular control bacteria were resuspended in DMEM prior to their lysis and total RNA extraction (*mir*Vana miRNA Isolation Kit, Ambion).

### Infection assays and harvesting of interacting cells

On the day of infection, host cell density was 1×10^6^ HeLa-S3 cells per well. Infection assays were performed as previously described [33], using an MOI of 5. Briefly, an according concentration of SL1344 cells was added to each well of pre-seeded HeLa-S3 cells. Subsequently, plates were centrifuged at 250 × g for 10 minutes in order to increase the infection efficiency. After 30 minutes of incubation at 37°C, the medium was replaced with freshly prepared culture medium containing 50 μg/ml of gentamicin to eliminate all adhered or suspended bacteria that did not successfully invade a host cell. Following a second incubation for 30 minutes, the medium was replaced with culture medium containing 10 μg/ml gentamicin, and the cells remained in the supplemented medium during further incubation.

Total RNA was isolated from non-interacting and infected HeLa-S3 cells after several points of time (0.5, 4, and 24 hours post infection, respectively). Prior to harvesting of the cells, all plates were washed with ice-cold phosphate buffered saline (PBS; Gibco). Then, the cells were solubilized with 0.25% trypsin (Gibco), subsequently washed with 1 ml of fresh culture medium per well, and centrifuged at 250 × g for 5 minutes. The obtained pellet was washed once with ice-cold PBS, followed by another centrifugation at 250 × g for 5 minutes, and resuspended in lysis buffer (*mir*Vana miRNA Isolation Kit, Ambion). Cells designated for transcription profiling of the early interaction were washed with ice-cold PBS and lysed, immediately after incubation in the culture medium containing 50 μg/ml gentamicin.

### Total RNA isolation and quality control

Isolation of total RNA was performed with the *mir*Vana miRNA Isolation Kit (Ambion) according to the manufacturer’s protocol for isolation of total RNA. The quality of total RNA was subsequently controlled on the Agilent 2100 Bioanalyzer. Twenty μg of total RNA from each interaction stage, 15 μg from naïve human host cells or 5 μg from extracellular *S. enterica* Typhimurium, respectively, were subjected to rRNA depletion for subsequent library construction (Table 2).

**Table 2:** **Summary of the rRNA depletion of total RNA from separately cultivated cells and interaction stages**

**Cells and interaction stages**	**Input amount of total RNA**	**Applied Ribo-Zero rRNA Removal Kit(s)**	**No. of reactions**
SL1344	5 μg	Gram-Negative Bacteria	1
HeLa-S3	15 μg	Human/Mouse/Rat	3
Early interaction*	50 μg	Human/Mouse/Rat	10
		Gram-Negative Bacteria	2
Mid-level Interaction	20 μg	Human/Mouse/Rat	4
		Gram-Negative Bacteria	1
Late Interaction	20 μg	Human/Mouse/Rat	4
		Gram-Negative Bacteria	1

*Two fifth of the depleted total RNA from early interacting cells were used for preparation of deepSuperSAGE and MACE libraries, respectively. The remaining fifth was kept for subsequent qRT-PCR validation.

Total RNA from cultivated HeLa-S3 and SL1344 cells was depleted with the correspondent Ribo-Zero rRNA Removal Kit, while RNA derived from infection assays was successively treated with both kit versions. The number of reactions for each type of kit reflects the depleted amount of rRNA in prokaryotic or eukaryotic samples. One reaction is sufficient to deplete 5 μg of the designated input total RNA.

### Construction of deepSuperSAGE dual 3’Seq libraries

In general, deepSuperSAGE library preparation was performed according to the high-throughput SuperSAGE protocol [39,40]. Both the polyadenylated as well as the non-polyadenylated RNA fraction of a given total RNA sample were used for construction of dual 3’Seq libraries to allow for genome-wide transcription profiling of the host cells and to assess the transcriptome from SL1344 cells. DNA fragments remaining in the total RNA isolate were digested by DNase I (Baseline-ZERO, Epicentre). Afterwards, total RNA was size-selected using Agencourt AMPure XP beads (Beckman Coulter) to exclude transcripts of less than ~150 nucleotides (Additional file 3). Ribosomal RNA depletion of the size-selected total RNA was subsequently carried out with the Ribo-Zero rRNA Removal Kits (Epicentre) ‘Gram-Negative Bacteria’ and ‘Human/Mouse/Rat’ (Table 2, Additional file 3). Isolates from infection assays were successively depleted, first with the kit suited for human total RNA, and then the kit for Gram-negative bacteria. The size-selected, rRNA-depleted total RNA was quantitatively precipitated, rehydrated and hybridized to Dynabeads Oligo dT_25_ (Invitrogen) for enrichment of polyadenylated transcripts. The enriched fraction was directly eluted from the beads, while the non-polyadenylated fraction was *in vitro* polyadenylated using the Poly(A) Tailing Kit (Ambion) preceded and followed by quantitative precipitation. The poly(A)^+^ and poly(A)^−^ RNA fractions were separately subjected to oligo(dT)-based reverse transcription using SuperScript III Reverse Transcriptase (Invitrogen) and an anchored, 5′ biotinylated oligo(dT) primer comprising the recognition site for EcoP15I. 3′ cDNA fragments were enriched through binding to streptavidin-coated paramagnetic beads (Dynabeads M-270Streptavidin, Invitrogen) subsequent to cleavage of 5′-CATG sites in the reverse-transcribed cDNAs by NlaIII (NEB). An adaptor comprising a known barcoding sequence and a second recognition site of EcoP15I was ligated to the enriched fragments using T4 DNA Ligase (Fermentas). The adaptor-ligated cDNA fragments were released from the beads via digestion by EcoP15I (NEB), end-repaired by KOD DNA Polymerase (Blunting High Kit, Toyobo), and subsequently ligated to a second barcoding adaptor using the T4 Ligase reagent provided with the Ligation high Ver. 2 Kit (Toyobo). Barcoding sequences of the adaptor-ligated cDNA fragments were extended during subsequent PCR-amplification employing Phusion Hot Start High-Fidelity DNA Polymerase (Fermentas) according to the manufacturer’s recommendations to incorporate the respective Illumina sequencing priming sites. Subsequent to PAGE purification, the amplified cDNA fragments were sequenced on the Illumina HiSeq2000 platform with single-end 50 base pair reads.

### Construction of MACE dual 3’Seq libraries

Massive analysis of cDNA ends (MACE) was essentially performed as previously described [17] with minor adjustments for dual 3’Seq. Preparation of total RNA was performed according to the procedure for preparation of deepSuperSAGE libraries, including separation of the polyadenylated from the non-polyadenylated transcripts, and *in vitro* polyadenylation of the latter fraction. Subsequent oligo(dT)-based reverse transcription of both fractions was performed under identical conditions, except for the use of an anchored, biotinylated oligo(dT) primer comprising a barcoding sequence instead of a recognition site for EcoP15I. Reverse-transcribed cDNAs were random-fragmented (Bioruptor, Diagenode) to an average size of approximately 200 nucleotides, and subsequently enriched for 3′ fragments through binding to streptavidin-coated paramagnetic beads (Dynabeads M-270 Streptavidin, Invitrogen). A second barcoding adaptor was ligated to the enriched 3′ fragments, and the adaptor-ligated cDNA fragments were PCR-amplified, PAGE-purified, and sequenced as described for deepSuperSAGE libraries.

### Construction of the operon-structured SL1344 transcriptome

In order to quantify the expression of polycistronically transcribed genes from the prokaryote via tag-based library preparation we combined the operon structure published by Ramachandran and colleagues [19] with the SL1344 genome from Kröger and co-workers [18]. The genomic locations of SL1344 transcripts with the respective operon structure were converted into BED format and subsequently used to extract the corresponding sequences of all generated entries via BEDtools [41] from the FASTA file of the genome. For polycistronically transcribed genes, the genomic sequence from the start of the first gene to the end of the last gene was extracted in full, thus generating only one entry for co-transcribed genes from a single operon in the new transcriptomic reference file.

### Processing of raw sequencing reads

Reads were sorted from bulked sequencing data according to respective barcodes. Barcodes and adaptor sequences were subsequently clipped from both ends of the sorted reads, and PCR duplicates identified by TrueQuant were excluded from the datasets. All bases detected as a low quality segment (FASTQ Sanger quality score below 16) indicated by the special Q2 score were trimmed from both sides of the reads to reduce false positive alignments due to poor sequencing results. Trimmed reads comprising less than 15 nucleotides were excluded from the datasets to further improve the reliability of annotation.

### Mapping, feature annotation, and quantification of processed reads

Processed reads were mapped to a combined reference comprising the operon-structured SL1344 reference transcriptome and the human transcriptome (RefSeq) as well as genome sequence hg19 from the UCSC table browser [42]. With respect to the dual 3’Seq approach a multi-step annotation procedure was employed to ensure the most reliable assignment of reads to the corresponding transcript. For both techniques, reads were first mapped against the trimmed transcriptome reference comprising the 3′ ends of all transcribed gene loci. The trimmed reference for deepSuperSAGE was generated via *in silico* digestion of the transcribed sequences by NlaIII, and comprised 26 base pair sequences corresponding to the last NlaIII site for each transcript, while the last 800 nucleotides of all transcript sequences were used for annotation of MACE reads. Unmapped reads were subsequently annotated to the full-length transcriptome reference. Still unmapped reads were poly(A)-trimmed and re-mapped to the full-length transcriptome, followed by annotation to the genomes. In each step reads mapping to the references of both organisms were excluded from re-annotation. DeepSuperSAGE reads were annotated with Bowtie allowing two mismatches, but reporting only those alignments with the lowest number of mismatches (−*v2 -strata -best*). MACE reads were mapped using the short read mapper Novoalign v2.07.13 (Novocraft Technologies) with default parameter settings.

### Median-based normalization

Annotated reads in each library were counted, and subsequently combined to three distinct expression matrices comprising reads from the poly(A)^−^ fraction of SL1344 and human host cells as well as the polyadenylated fraction of the host cells, respectively. The three matrices were separately processed with DESeq [43]. Briefly, a normalization factor for each library was calculated as the median of ratios between the counts in the library and a pseudo reference, which is defined as the geometric mean of each gene within all libraries. In comparison to normalization through conversion of read numbers to tags per million (TPM), median-normalization accounts for extreme cases, where a few highly differentially expressed genes reduce the amount of sequencing reads for other transcripts exceptionally in one of the compared libraries. It might therefore be interpreted as an outlier-independent average sample, which is especially important with respect to the varying amount of prokaryotic transcripts in the different interaction stages.

### Differential expression and statistical testing

Statistical significance was determined by *χ*^2^ tests based on the original read count [44], and differential expression in the course of infection by pair-wise comparison of the normalized read numbers. For calculation of the *χ*^2^–values, original library sizes were adjusted with a median-based normalization factor for each comparison to account for potential outliers. The calculated p-values were subsequently adjusted for multiple testing with the procedure described by Benjamini and Hochberg [45]. Normalized read counts of zero were adjusted to 0.05 to allow for calculation of fold changes even if a given tag was only present in one of the compared libraries.

### Evaluation of dual 3’Seq expression ratios of selected mRNAs and their degradation intermediates by probe-based qRT-PCR

Quantification accuracy of dual 3’Seq coupled either to deepSuperSAGE or alternatively to MACE was assessed with the remaining 10 μg DNase-treated and rRNA-depleted total RNA from library preparation of the early interaction stage (see Table 2). The poly(A)^+^ and poly(A)^−^ fractions corresponding to 10 μg of original total RNA were filled up to 100 μl volume, respectively, to restore the original equilibrium between both fractions. 23 mRNAs previously confirmed as expressed during early interaction with at least one of both library preparation techniques were subsequently quantified by real-time PCR on the StepOne Real-Time PCR System (Applied Biosystems). Amplification reactions were carried out in 12 μl volume using the One Step PrimeScript RT-PCR Kit (Perfect Real Time) from TaKaRa complemented by specific forward and reverse primers in a final concentration of 200 nM each and a dual-labeled (5′ FAM reporter and 3′ BHQ-1, DDQ-1, or TQ2 quencher) probe in a final concentration of 133 nM (Additional file 4: Table S3). The equivalent of 100 ng total RNA prior to rRNA depletion and fractionation into the poly(A)^+^ and poly(A)^−^ RNA (one μl out of the 100 μl volume for each fraction) served as template for each reaction. Initial reverse transcription, denaturation and subsequent cycling followed the recommendation of the manufacturer, with a slightly prolonged annealing and elongation step. Subsequent to reverse transcription for 5 minutes at 42°C initial denaturation was performed at 95°C for 10 seconds, followed by 40 cycles of 5 seconds at 95°C and 30 seconds at 60°C. Target mRNAs were quantified in triplicates for each template to calculate the ∆C_t_s between the target mRNAs and their degradation intermediates using arithmetic means. Normalization using a reference gene was not possible, due to the varying abundance of degradation intermediates, and fractionation into the poly(A)^+^ and poly(A)^−^ RNA impeded the use of spike-ins for this purpose. For comparison of the ∆C_t_ values with the expression ratios identified by dual 3’Seq, human reads from the poly(A)^+^ and poly(A)^−^ libraries generated with the two different 3′ profiling techniques were separately TPM-normalized and log_2_-transformed. The ratios between the functional, polyadenylated mRNAs and their non-polyadenylated degradation intermediates were subsequently determined analogous to the ∆C_t_ calculation employed for qRT-PCR.

### Quantitative real-time PCR-based validation of time-dependent expression profiles determined by dual 3’Seq using deepSuperSAGE

Time-dependent expression of candidate genes from the prokaryotic as well as eukaryotic cells was validated using three independent biological replicates that were prepared in the same way as described above including DNase I digestion, but omitting the size selection and rRNA depletion step. Subsequent to quantification of total RNA (Qubit, Life Technologies) the isolates were reverse-transcribed with SuperScript III Reverse Transcriptase (Invitrogen). All amplification reactions were carried out in 12 μl volume with the 5x HOT MOLPol EvaGreen qPCR Mix (ROX) from Molegene, complemented by the respective forward and reverse primers in a final concentration of 250 nM each (Additional file 5: Table S4). Initial denaturation was performed at 95°C for 15 minutes, followed by 40 cycles of 15 seconds at 95°C, 20 seconds at 65°C and 30 seconds at 72°C. A final elongation step at 72°C for 5 minutes allowed the polymerase to complete all unfinished strands. Subsequently, a melting curve analysis was performed to verify exclusive amplification of the expected products. Reverse-transcribed cDNA corresponding to 40 ng total RNA in each amplification reaction on the StepOne Real-Time PCR System was used for quantification of the eukaryotic expression patterns, while the cDNA equivalent of 20 ng total RNA from non-interacting pathogen cells and 100 ng total RNA from interacting pathogen cells served for validation of the prokaryotic expression profiles.

Relative transcript abundances between the different points of time were calculated according to the ∆∆Ct method using the geometric mean of three and four (host and pathogen cells, respectively) previously determined reference genes. All target and reference mRNAs were quantified in duplicates for all biological replicates, respectively. The arithmetic mean of each duplicate was then used to calculate ∆∆Ct values between the samples. *GAPD*, *B2M* and *RPL13A* were employed as reference genes for normalization of the transcript abundances in host cells [46], while seven candidate reference genes from the pathogen were previously screened for their relative expression stability across the different replicates and interaction stages according to Bestkeeper [47], NormFinder [48], and GeNorm [46].

### Supporting data

The adaptor-sorted and PCR duplicate-free raw sequencing data is available at GEO (Accession number: GSE61730) along with the three expression matrices that contain the median-normalized values for the respective samples after full processing of the raw data. Raw read counts are additionally provided for the two libraries generated by MACE.

## Additional files

Additional file 1: Table S1. Annotation statistics of dual 3’Seq libraries prepared with deepSuperSAGE (top) and MACE (bottom). The number of reads mapped to human, SL1344 or both references in each step of the sequential annotation procedure is given for sense and antisense (AS) annotated transcripts, respectively. Pre-processed reads were first annotated to a trimmed and full-length transcriptome reference, and finally aligned to the complete genome of both organisms. The number of reads mapped to intronic/intergenic regions is additionally listed for the genomic annotation step. Additional file 2: Table S2. List of clustered, plasmid-encoded transcripts from SL1344. Gene symbols are listed along with the respective log_2_ fold changes for each of the four clusters in a separate worksheet. Additional file 3:
**Supplementary notes and figures.**
Additional file 4: Table S3. List of targeted mRNAs along with the respective primer and probe sequences used for evaluation of dual 3’Seq quantification accuracy. Additional file 5: Table S4. Primer sequences used for qRT-PCR quantification of selected mRNAs from host and pathogen cells.
