# Supplementary material for: Dual 3’Seq using deepSuperSAGE uncovers transcriptomes of interacting Salmonella enterica Typhimurium and human host cells
Source: BMC Genomics. 2015 Apr 19;16(1):323. doi: 10.1186/s12864-015-1489-1 (PMC4480994; doi:10.1186/s12864-015-1489-1)
Supplement: Additional file 1: Table S1. — Annotation statistics of dual 3’Seq libraries prepared with deepSuperSAGE (top) and MACE (bottom). The number of reads mapped to human, SL1344 or both references in each step of the sequential annotation procedure is given for sense and antisense (AS) annotated transcripts, respectively. Pre-processed reads were first annotated to a trimmed and full-length transcriptome reference, and finally aligned to the complete genome of both organisms. The number of reads mapped to intronic/intergenic regions is additionally listed for the genomic annotation step. [file 12864_2015_1489_MOESM1_ESM.docx]

**Additional file 1: Table S1** | Annotation statistics of dual 3’Seq libraries prepared with deepSuperSAGE (top) and MACE (bottom). The number of reads mapped to human, SL1344 or both references in each step of the sequential annotation procedure is given for sense and antisense (AS) annotated transcripts, respectively. Pre-processed reads were first annotated to a trimmed and full-length transcriptome reference, and finally aligned to the complete genome of both organisms. The number of reads mapped to intronic/intergenic regions is additionally listed for the genomic annotation step.

|  |  | Trimmed transcriptome reference | | | | | |  | Full-length transcriptome reference | | | | | | |  | Genome reference | | | | | |
| --- | --- | --- | --- | --- | --- | --- | --- | --- | --- | --- | --- | --- | --- | --- | --- | --- | --- | --- | --- | --- | --- | --- |
|  |  | Sense Human | Sense SL1344 | AS Human | AS SL1344 | Sense Both | AS Both |  | | Sense Human | Sense  SL1344 | AS Human | AS  SL1344 | Sense Both | AS Both |  | Sense Human | Sense SL1344 | AS Human | AS  SL1344 | Intronic/ Intergenic Human | Intronic/ Intergenic SL1344 |
|  |  |  |  |  |  |  |  |  | |  |  |  |  |  |  |  |  |  |  |  |  |  |
| Poly(A)^+^ | Cultivated HeLa | 215,877 | 38 | 5,101 | 0 | 11 | 0 |  | | 690,716 | 117 | 4,838 | 16 | 140 | 18 |  | 9,337 | 7 | 96 | 2 | 40,225 | 87 |
|  | Cultivated SL1344 | 821 | 4,130 | 361 | 0 | 26 | 1 |  | | 2,925 | 49,077 | 791 | 3,070 | 478 | 25 |  | 2 | 1,285 | 3 | 328 | 1,242 | 9,516 |
|  | Early interaction | 775,039 | 22 | 2,143 | 3 | 34 | 0 |  | | 2,808,463 | 32 | 10,941 | 157 | 199 | 12 |  | 61,258 | 0 | 1,024 | 0 | 182,556 | 1 |
|  | Mid-level interaction | 900,565 | 146 | 10,974 | 3 | 86 | 0 |  | | 2,367,763 | 73 | 24,197 | 1,389 | 284 | 24 |  | 44,410 | 1 | 511 | 0 | 163,033 | 8 |
|  | Late interaction | 69,508 | 12 | 2,774 | 5 | 2 | 0 |  | | 182,166 | 30 | 4,911 | 459 | 80 | 7 |  | 2,606 | 0 | 34 | 0 | 14,438 | 3 |
|  |  |  |  |  |  |  |  |  | |  |  |  |  |  |  |  |  |  |  |  |  |  |
| Poly(A)^-^ | Cultivated HeLa | 513,553 | 3,188 | 360 | 3 | 33 | 0 |  | | 1,441,721 | 473 | 56,749 | 843 | 2,265 | 117 |  | 46,764 | 0 | 359 | 4 | 424,382 | 49 |
|  | Cultivated SL1344 | 3,012 | 427,943 | 758 | 8 | 1,406 | 2 |  | | 16,203 | 2,578,869 | 4,560 | 209,018 | 5,184 | 423 |  | 8 | 219,768 | 8 | 86,736 | 8,717 | 671,836 |
|  | Early interaction | 1,141,619 | 7,280 | 2,723 | 22 | 321 | 0 |  | | 4,477,275 | 2,382 | 163,302 | 57,199 | 10,310 | 841 |  | 394,943 | 73 | 1,455 | 12 | 1,250,650 | 585 |
|  | Mid-level interaction | 1,261,148 | 13,262 | 7,788 | 11 | 308 | 0 |  | | 3,128,988 | 4,813 | 819,208 | 113,229 | 8,282 | 708 |  | 115,468 | 37 | 584 | 8 | 780,340 | 1,280 |
|  | Late interaction | 1,052,272 | 13,612 | 1,757 | 8 | 281 | 0 |  | | 3,197,253 | 6,004 | 460,575 | 97,297 | 10,999 | 548 |  | 170,162 | 143 | 421 | 21 | 727,989 | 616 |
|  |  |  |  |  |  |  |  |  | |  |  |  |  |  |  |  |  |  |  |  |  |  |

|  | Trimmed transcriptome reference | | | | | |  | Full-length transcriptome reference | | | | | |  | Genome reference | | | | | |
| --- | --- | --- | --- | --- | --- | --- | --- | --- | --- | --- | --- | --- | --- | --- | --- | --- | --- | --- | --- | --- |
|  | Sense Human | Sense SL1344 | AS Human | AS SL1344 | Sense Both | AS Both |  | Sense Human | Sense  SL1344 | AS Human | AS  SL1344 | Sense Both | AS Both |  | Sense Human | Sense SL1344 | AS Human | AS SL1344 | Intronic/ Intergenic Human | Intronic/ Intergenic SL1344 |
| Early int. Poly(A)^+^ | 6,439,866 | 886 | 111,130 | 341 | 2,054 | 335 |  | 2,208,954 | 234 | 121,089 | 363 | 162 | 48 |  | 242,067 | 0 | 8,818 | 1 | 766,292 | 2 |
| Early int. Poly(A)^-^ | 5,772,379 | 9,268 | 412,585 | 3,686 | 14,015 | 1,481 |  | 647,582 | 3,199 | 154,645 | 12,764 | 1,682 | 175 |  | 316,062 | 135 | 5,686 | 26 | 1,387,888 | 148 |
