# Supplementary material for: Dual 3’Seq using deepSuperSAGE uncovers transcriptomes of interacting Salmonella enterica Typhimurium and human host cells
Source: BMC Genomics. 2015 Apr 19;16(1):323. doi: 10.1186/s12864-015-1489-1 (PMC4480994; doi:10.1186/s12864-015-1489-1)
Supplement: Additional file 3: — Supplementary notes and figures. [file 12864_2015_1489_MOESM3_ESM.docx]

# Supplementary Notes and Figures

# Supplementary Note 1 – Total RNA size selection using AMPure XP

Digestion of synthesized cDNA by the anchoring enzyme NlaIII is a crucial step in formation of deepSuperSAGE tags, because the 5’ ends of future tags are determined by the cleavage site. This implies that a tag from a reverse-transcribed cDNA is obtained, given that the restriction site is present at least once, and that cleavage by the anchoring enzyme was successful. The recognition site of NlaIII corresponds to the palindromic sequence 5’-CATG-3’, and consequently cleavage occurs at an average of 256 nucleotides distance. Cleavage of a 100 nucleotide cDNA fragment therefore only occurs with a statistical chance of approximately 40%. In order to preserve small RNA transcripts for separate analysis, a size-selective cut-off of the total RNA was performed using the Agencourt AMPure XP system (Beckman Coulter). The system was originally designed for PCR purification, and comprises a relatively accurate, size-selective cut-off to remove primer dimers and other low molecular weight contaminants by solid-phase reversible immobilization (SPRI) coupled to magnetic beads [[1](#_ENREF_1)]. Briefly, DNA is selectively bound to the SPRI beads through addition of a 20% polyethylene glycol (PEG) and 2.5 M NaCl buffer. Unbound fragments can subsequently be removed with the supernatant. In this process, size selection of unwanted lower molecular weight DNA fragments is adjusted through the volume of buffer that is added to the reaction, changing the final concentration of PEG in the resulting mixture and altering the size range of bead-bound fragments. The recommended ratio for PCR-purification is 1.8 volumes of beads for 1 volume of the sample, respectively. We tested the system for RNase activity and performed a titration using different ratios of bead and sample volumes (1.8, 1.5, 1.2, 0.9, and 0.6, respectively) to find an adequate lower molecular weight limit for size selection of total RNA prior to library preparation. For that purpose, a low range ssRNA ladder (NEB) and a mixture of the RNA 6000 Nano as well as small RNA ladder (Agilent) were subjected to purification using the above ratios, and subsequently analyzed on the Agilent 2100 Bioanalyzer.

Electrophoretic profiles of the mixed ladder (comprising the Nano and small RNA ladder) before and after treatment exhibit varying numbers of peaks, which is consistent with the expected tendency of size selection for the respective ratios (**Supplementary Fig. 1a**). The Nano ladder comprises six peaks at 200, 500, 1,000, 2,000, 4,000, as well as 6,000 nucleotides, and only the latter two remain unaffected after purification using the 0.6 ratio, which contains the lowest amount of PEG in the reaction mixture. Increment to a ratio of 0.9 leaves two more peaks unaffected, indicating that the size-selective ratio is very dynamic in the region below one. On the other hand, electrophoretic profiles of the samples treated with a ratio of 1.8 and 1.5 are very similar, also in the region of the small RNA ladder (up to 150 nucleotides). Compared to the untreated Nano ladder, only the very first peak at 200 nucleotides is affected by purification with the 1.8 ratio, proving that this system can


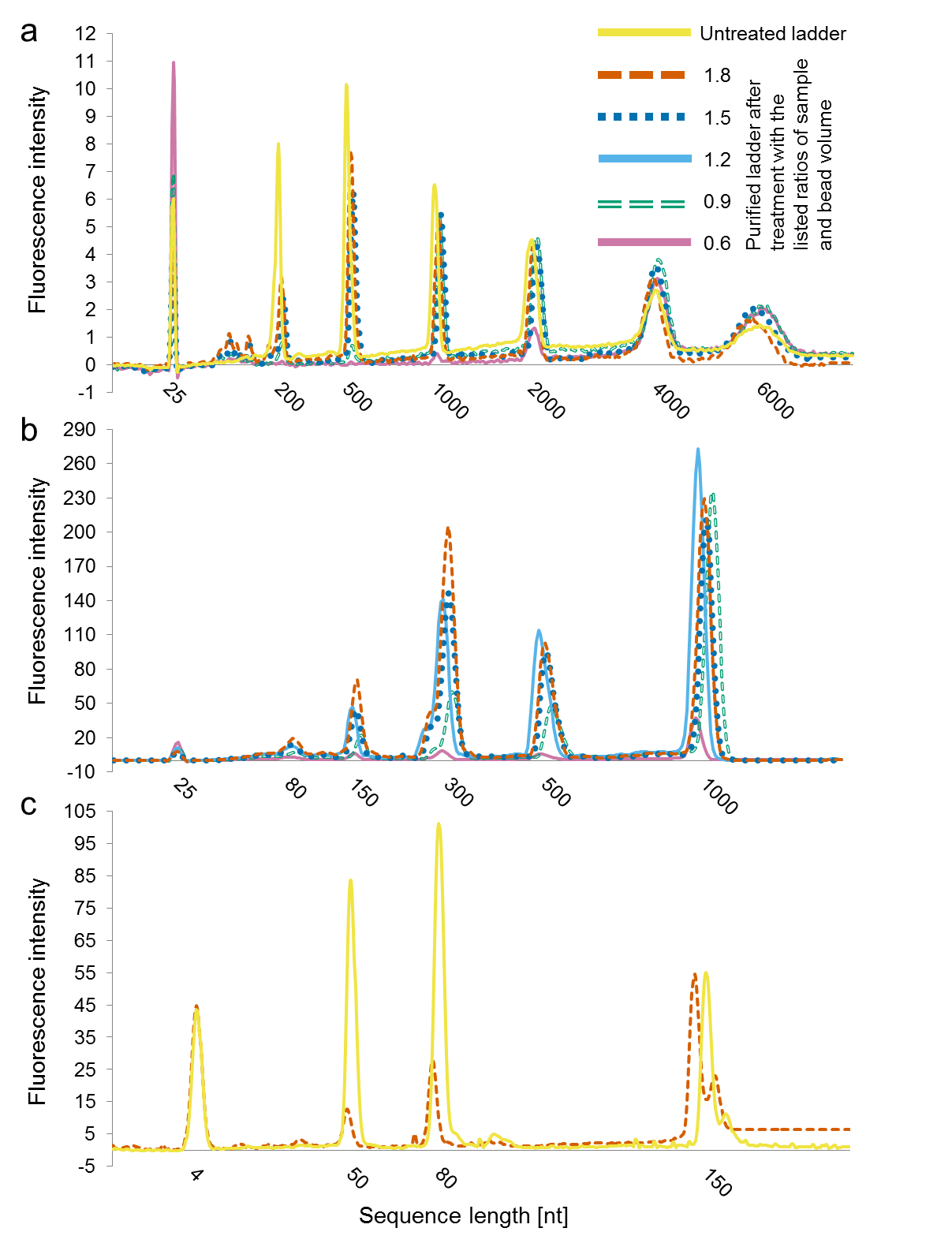


Supplementary Figure 1 | Comparison of electrophoretic profiles from purified and untreated ladders. Fluorescence intensity (FU) is plotted against the fragment size of the transcripts in nucleotides (nt). Respective data was obtained with the RNA 6000 Pico kit using a mixed ladder comprising the Nano and small RNA ladder from Agilent (**a**), as well as the RNA 6000 Nano (**b**) and Small RNA (**c**) kit using a low range ladder from NEB. Peaks below 50 nucleotides represent internal markers that are necessary for calibration. For reasons of comparability untreated total RNA (yellow) is shown compressed.

be used for the desired size selection of total RNA in a region of approximately 200 nucleotides. To determine the respective cut-off in more detail, we employed a low-range ladder comprising RNA molecules peaking at 50, 80, 150, 300, 500, and 1,000 nucleotides. Additionally to electrophoresis in a region corresponding to mRNA (**Supplementary Fig. 1b**), the purified and untreated low-range ladders were analyzed with higher resolution using a small RNA chip (**Supplementary Fig. 1c**). The peaks at 1,000, 500 and 300 nucleotides were not affected by purification with the recommended ratio, while the first two peaks at 50 and 80 nucleotides are significantly reduced. The abundance of the 150 nucleotide fragment in between is slightly reduced, indicating that the lower molecular weight limit for size selection is almost reached. Taken together, the electrophoretic profiles show that purification with the recommended ratio results in a significant depletion of transcripts below 100 nucleotides, while those around 200 are mostly, and the ones above 300 nucleotides completely retained. Consequently, the AMPure XP system is perfectly suited for the desired size-selective cut‑off of RNA fragments prior to library preparation.

Although the applied size-selection is recommended for deepSuperSAGE library preparation to preserve small RNA sequences for separate analysis, it is not mandatory for generation of MACE libraries. Size-selection of total RNA for MACE was solely performed to preserve the comparability of both datasets.

# Supplementary Note 2 – Ribosomal RNA depletion

To further decrease the necessary sequencing depth for simultaneous characterization of both transcriptomes from interacting SL1344 and human host cells without sacrificing relevant information, we tested four different commercially available ribosomal RNA (rRNA) depletion kits, and finally employed the best-performing kit for dual 3’Seq library preparation. An efficient depletion of ribosomal RNA permits more extensive transcriptome profiling of the poly(A)^−^ RNA fraction due to the lower sequencing depth, which is required to obtain a sufficient coverage for all other transcripts [[2](#_ENREF_2)].

DNase-treated total RNA from the competent *Escherichia coli* (*E. coli*) strain DH5α K12 (New England Biolabs) served to test the efficiency of four different commercial rRNA depletion methods. These can be broadly classified into the two groups of hybridization-based (MICROBExpress, Ribominus, Ribo-Zero) and phosphorylation-dependent (mRNA-ONLY) techniques (**Table 1**). Even though different kits are necessary for rRNA removal in pro- and eukaryotes, only kits for depletion of prokaryotic rRNA were tested, since the performance of two corresponding kits from the same manufacturer is expected to be similar, as the underlying principles are preserved.

*E. coli* cells from a glycerol stock stored at -80 °C were seeded onto LB plates containing 100 µg/ml ampicillin. These plates were aerobically incubated at 37 °C overnight. A single colony from one of these plates served to inoculate LB medium containing 100 µg/ml ampicillin for suspension culture. The suspension culture was aerobically cultivated at 37 °C using a heated shaker tray, until an $\mathrm{OD}_{600}$ of 0.5 was reached. Afterwards, the bacteria were harvested by centrifugation and resuspended in lysis buffer RLT (RNeasy Kit, Qiagen). Isolation of total RNA from *E. coli* was carried out with the RNeasy Mini Kit (Qiagen) according to the protocol for purification of total RNA from plant cells, tissues and filamentous fungi. The re-suspended cells were disrupted by addition of liquid nitrogen, followed by grinding with sterilized mortar and pestle, and further homogenization using QIAshredder homogenizers (Qiagen). The application of all four rRNA removal methods followed the instructions of the manufacturers, and all depletion reactions were carried out with the maximal input amount of total RNA for reasons of comparability. Subsequent evaluation of the rRNA depletion efficiency was based on data from electrophoretic assays carried out on the Agilent 2100 Bioanalyzer.

| **References** | [[3](#_ENREF_3), [4](#_ENREF_4)] | [[3-5](#_ENREF_3)] | [[6-8](#_ENREF_6)] | [[9](#_ENREF_9), [10](#_ENREF_10)] |
| --- | --- | --- | --- | --- |
| **Input amount** | 10 µg | 10 µg | 10 µg | 5 µg |
| **Targeted rRNA** | 23S and 16S rRNA | 23S and 16S rRNA | 23S and 16S rRNA | 23S, 16S, and  5S rRNA |
| **Description of the respective**  **rRNA removal method** | RNA transcripts with a 5´ monophosphate are digested via Terminator 5´-Phosphate-Dependent Exonuclease. | A bridging nucleic acid is hybridized, first with the ribosomal target RNA, and then with a capture nucleic acid, which is covalently bound to a paramagnetic bead. | Biotinylated locked nucleic acid (LNA) probes are hybridized with the ribosomal target RNA, and subsequently bound to streptavidin-coated, paramagnetic beads. | Affinity-tagged, full-length antisense rRNA baits are annealed to the correspondent target rRNA, followed by an affinity-based separation via Ribo-Zero Microspheres. |
| **Company** | Epicentre | Ambion | Invitrogen | Epicentre |
| **Brand of rRNA**  **Table 1** \| The different rRNA removal kits tested for this study. A brief description of the depletion method and the designated target rRNA is listed along with the respective manufacturer. The applied total RNA input corresponds to exactly one reaction of the respective kit.  **removal kit** | mRNA-ONLY  Prokaryotic mRNA Isolation Kit | MICROBExpress  Bacterial mRNA Enrichment Kit | Ribominus  Bacteria Transcriptome Isolation Kit | Ribo-Zero  rRNA Removal Kit  (Gram-Negative Bacteria) |

The high abundance of rRNA transcripts is largely reduced regardless of the employed removal method (**Supplementary Fig. 2**). The well-defined peaks of the 16S and 23S rRNAs, present in the untreated sample at about 1,500 and 2,900 nucleotides, are almost absent in all treated total RNA samples. The electrophoretic profile of re-eluted rRNA from the Ribominus treatment is similar to the one of the untreated total RNA sample (not shown). Compared to each other, the different rRNA removal methods produce distinct electrophoretic profiles, indicating varying efficiencies of rRNA depletion.


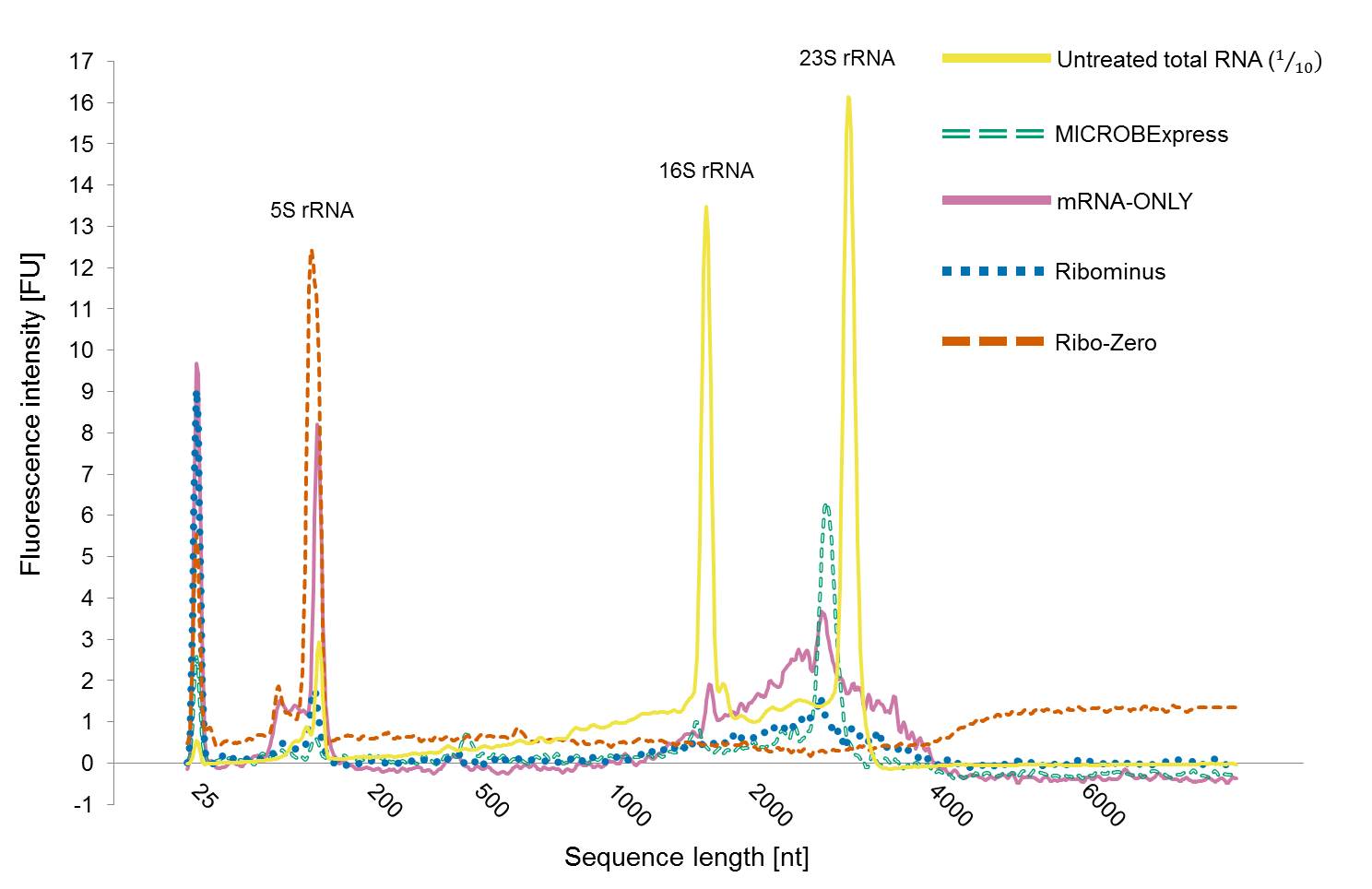


**Supplementary Figure 2** | Electrophoretic profile of *E. coli* total RNA before and after depletion of ribosomal RNA. The electropherogram depicts rRNA-depleted samples after treatment with the MICROBExpress (green), mRNA-ONLY (purple), Ribominus (blue), and Ribo-Zero kits (vermillion) as well as the untreated total RNA (yellow) compressed by a factor of 10 for reasons of comparability. Please consult Figure 1 for further details.

The 2,900 nucleotide region is significantly depleted in the profiles of Ribominus and Ribo-Zero-treated total RNA, while treatment with MICROBExpress leaves a distinct peak at approximately 2,600 nucleotides indicating that partially degraded 23S rRNA fragments remained in solution. mRNA-ONLY treated total RNA also retained a considerable amount of transcripts in that region and furthermore a broad distribution of transcripts ranging from approximately 4,000 down to 1,200 nucleotides. In contrast, 16S rRNA was efficiently depleted by all other kits. Regarding the size range of both 16S and 23S rRNA, depletion using Ribo-Zero results in the lowest fluorescence of all treatments. Conversely, Ribo-Zero-treated total RNA displays the highest fluorescence at about 120 nucleotides, although 5S rRNAs are also targeted by the employed full-length antisense baits.

Ultimately, we used the Ribo-Zero rRNA Removal Kits (Gram-Negative Bacteria and Human/Mouse/Rat) for rRNA depletion of total RNA from both separately cultivated and interacting HeLa-S3 and SL1344 cells. Apart from the superior 16S and 23S rRNA depletion efficiency compared to the other hybridization-based techniques, the full-length antisense rRNA baits also target partially degraded rRNA fragments that lack the presence of an annealing site for the relatively limited capture probes of the other kits. The less efficient 5S rRNA removal is not of interest, since total RNA of human host and pathogen cells was size-selected prior to the depletion of rRNAs. The phosphorylation-dependent exonuclease treatment of the mRNA-ONLY kit is not suited for quantitative transcriptome profiling, because of the large amount of remaining, partially degraded fragments that likely inflate library preparation. The broad distribution of transcripts in both the 2,900 and 1,500 nucleotide region of mRNA‑ONLY-treated total RNA shows that digestion of 16S and 23S rRNA by Terminator 5´-Phosphate-Dependent Exonuclease is incomplete. Since this region comprises most processed mRNAs, size selection of these fragments is not feasible.

# References

1. Fisher S, Barry A, Abreu J, Minie B, Nolan J, Delorey TM, Young G, Fennell TJ, Allen A, Ambrogio L et al: A scalable, fully automated process for construction of sequence-ready human exome targeted capture libraries. Genome Biol 2011, 12(1):R1.

2. Raz T, Kapranov P, Lipson D, Letovsky S, Milos PM, Thompson JF: Protocol dependence of sequencing-based gene expression measurements. PLoS One 2011, 6(5):e19287.

3. Mettel C, Kim Y, Shrestha PM, Liesack W: Extraction of mRNA from soil. Appl Environ Microbiol 2010, 76(17):5995-6000.

4. He S, Wurtzel O, Singh K, Froula JL, Yilmaz S, Tringe SG, Wang Z, Chen F, Lindquist EA, Sorek R et al: Validation of two ribosomal RNA removal methods for microbial metatranscriptomics. Nat Methods 2010, 7(10):807-812.

5. Mendoza LG, Moturi S, Setterquist R, Whitley JP: Methods and compositions for depleting abundant RNA transcripts. In. United States; 2006.

6. Braasch DA, Corey DR: Locked nucleic acid (LNA): fine-tuning the recognition of DNA and RNA. Chem Biol 2001, 8(1):1-7.

7. McTigue PM, Peterson RJ, Kahn JD: Sequence-dependent thermodynamic parameters for locked nucleic acid (LNA)-DNA duplex formation. Biochemistry 2004, 43(18):5388-5405.

8. Ruan Y, Le Ber P, Ng HH, Liu ET: Interrogating the transcriptome. Trends Biotechnol 2004, 22(1):23-30.

9. Su C, Sordillo LM: A simple method to enrich mRNA from total prokaryotic RNA. Mol Biotechnol 1998, 10(1):83-85.

10. Sooknanan RR: Methods, compositions, and kits for generating rRNA-depleted samples or isolating rRNA from samples. In. United States; 2011.
