# Supplementary material for: Dual 3’Seq using deepSuperSAGE uncovers transcriptomes of interacting Salmonella enterica Typhimurium and human host cells
Source: BMC Genomics. 2015 Apr 19;16(1):323. doi: 10.1186/s12864-015-1489-1 (PMC4480994; doi:10.1186/s12864-015-1489-1)
Supplement: Additional file 4: Table S3. — List of targeted mRNAs along with the respective primer and probe sequences used for evaluation of dual 3’Seq quantification accuracy. [file 12864_2015_1489_MOESM4_ESM.docx]

| **Gene symbol^*^** | **Alias** | **Accession number^†^ / localization^*^** | **Primer / probe** | **Sequence** | **Amplicon length** |
| --- | --- | --- | --- | --- | --- |
| *ACTB^+^* | Actin Beta | NM_001101.3 7p22 | Forward | CTGGAACGGTGAAGGTGACA | 133 |
|  |  |  | Reverse | TTCCTGTAACAACGCATCTCA |  |
|  |  |  | Probe | CCGAGGACTTTGATTGCACATTGTTGT |  |
| *APOD* | Apolipo-protein D | NM_001647.3 3q29 | Forward | CATCCAGGCCAACTACTCACT | 100 |
|  |  |  | Reverse | GGCTTCACCTTCGATTTGATT |  |
|  |  |  | Probe | CAGTTCCATCAGCTCTCAACTCCTGGTTTA |  |
| *B2M^+^* | Beta-2-microglobulin | NM_004048.2 15q21-q22.2 | Forward | TGAGTGCTGTCTCCATGTTTG | 90 |
|  |  |  | Reverse | TCTCTGCTCCCCACCTCTAA |  |
|  |  |  | Probe | ATCTGAGCAGGTTGCTCCACAGGT |  |
| *BMP2* | Bone morphogenetic protein 2 | NM_001200.2 20p12 | Forward | GGGTGGAATGACTGGATTGT | 106 |
|  |  |  | Reverse | TGGCATGATTAGTGGAGTTCAG |  |
|  |  |  | Probe | ATTCTCCGTGGCAGTAAAAGGCGTGATAC |  |
| *CDK1* | Cyclin-dependent kinase 1 | NM_001170406.1 NM_001170407.1 NM_001786.4 NM_033379.4 10q21.2 | Forward | TCAGACTAGAAAGTGAAGAGGAAGG | 140 |
|  |  |  | Reverse | CTCAAAGATGAGATATAACCTGGAA |  |
|  |  |  | Probe | CCAAATATAGTCAGTCTTCAGGATGTG |  |
| *CENPA* | Centromere protein A | NM_001809.3 2p23.3 | Forward | GCCGCCTGGCAAGAGAAATA | 159 |
|  |  |  | Reverse | GAGTAACTCGGCCTGCATGT |  |
|  |  |  | Probe | GCCCAGGCCCTATTGGCCCT |  |
| *CHAC1* | ChaC, cation transport regulator homolog 1 (*E. coli*) | NM_001142776.1 NM_024111.3 15q15.1 | Forward | TGGATTTTCGGGTACGGCTC | 141 |
|  |  |  | Reverse | ACGGCCAGGCATCTTGTC |  |
|  |  |  | Probe | CCGCCGTTTCTGGCAGGGAG |  |
| *CLIC3* | Chloride intracellular channel 3 | NM_004669.2 9q34.3 | Forward | CTCAAGGGCGTACCTTTCAC | 130 |
|  |  |  | Reverse | GCAGCGTGTCTGTCTTGG |  |
|  |  |  | Probe | TGCCCATCCTGCTCTATGACAGCGAC |  |
|  |  |  |  |  |  |
|  |  |  |  |  |  |
| *CORIN* | Corin, serine peptidase | NM_001278585.1 NM_006587.3 4p13-p12 | Forward | TAAGCTGCAAGAGGGAGAGGT | 96 |
|  |  |  | Reverse | CCAGCACATATCATCCGAGT |  |
|  |  |  | Probe | CAGTCCTACTTTGACATGAAGACCATCACC |  |
| *CTH* | Cystathionase (cystathionine gamma-lyase) | NM_001190463.1 NM_001902.5 NM_153742.4 1p31.1 | Forward | TATGGATGATGTGTATGGAG | 126 |
|  |  |  | Reverse | GTTTCTGGTGTAATTGCTG |  |
|  |  |  | Probe | CAGGCAAGTGGCATCTGAATTTGGA |  |
| *CTNNB1* | Catenin (cadherin-associated protein), beta 1.88 kDa | NM_001098209.1 NM_001098210.1 NM_001904.3 3p21 | Forward | CTAAATACCATTCCATTGTTTGTG | 129 |
|  |  |  | Reverse | TCCCTCAGCTTCAATAGCTTC |  |
|  |  |  | Probe | CTGCTTTATTCTCCCATTGAAAACATCCA |  |
| *FST^++^* | Follistatin | NM_006350.3 NM_013409.2 5q11.2 | Forward | CAGTAAGTCGGATGAGCCTGTCT | 74 |
|  |  |  | Reverse | CAGCTTCCTTCATGGCACACT |  |
|  |  |  | Probe | TGCCAGTGACAATGCCACTTATGCCA |  |
| *HJURP* | Holliday junction recognition protein | NM_001282962.1 NM_001282963.1 NM_018410.4 2q37.1 | Forward | GCAGCGGCTGATAGAGAAGT | 93 |
|  |  |  | Reverse | CTCAATCCCTGTGGCGTCTC |  |
|  |  |  | Probe | CCCTTCGAGGACACCCCGGT |  |
| *HMOX1* | Heme oxygenase (decycling) 1 | NM_002133.2 22q12 | Forward | AGCAACAAAGTGCAAGAT | 110 |
|  |  |  | Reverse | AGAAAGCTGAGTGTAAGGAC |  |
|  |  |  | Probe | CCAGAGGGAAGCCCCCACTCAA |  |
| *ITGB3* | Integrin, beta 3 (platelet glycoprotein IIIa, antigen CD61) | NM_000212.2 17q21.32 | Forward | CCTGTCCCTCATCCATAGCAC | 102 |
|  |  |  | Reverse | GCAGCCAAGAGGTAGAAGGTAA |  |
|  |  |  | Probe | ATGGATGGATACAGCACACCAAGGCAC |  |
| *KRT15* | Keratin 15 | NM_002275.3 17q21.2 | Forward | CAGATCCAGGGGCTCATT | 114 |
|  |  |  | Reverse | CTCCAGCCGTGTCTTTATGTC |  |
|  |  |  | Probe | AGCAGCATCTTGTACTCCTGGTTCTGAGC |  |
|  |  |  |  |  |  |
|  |  |  |  |  |  |
| *MARS* | Methionyl-tRNA synthetase | NM_004990.3 12q13 | Forward | CAGCAACAGAGACCAAGG | 108 |
|  |  |  | Reverse | CCAGCGGTAGATGTCAGCA |  |
|  |  |  | Probe | TCTGCGACAAGTACCACATCATCCA |  |
| *MNX1* | Motor neuron and pancreas homeobox 1 | NM_001165255.1 NM_005515.3 7q36 | Forward | CCCAGGTGAAGATTTGGTTC | 93 |
|  |  |  | Reverse | TTCTGTTTCTCCGCTTCCTG |  |
|  |  |  | Probe | ACGCAGCAAAAAGGCCAAAGAGCA |  |
| *PCNA* | Proliferating cell nuclear antigen | NM_002592.2 NM_182649.1 20p13-p12.3 | Forward | GCCATATTGGAGATGCTGTTG | 127 |
|  |  |  | Reverse | GCTTCCTCCTCTTTATCGACATT |  |
|  |  |  | Probe | AAGTGGAGAACTTGGAAATGGAAACATTA |  |
| *RPL13A^+^* | Ribosomal protein L13a | NM_001270491.1 NM_012423.3 NR_003932.2 NR_004844.1 NR_026712.1 NR_073024.1 19q13.3 | Forward | TTGAGGACCTCTGTGTATTTGTCAA | 126 |
|  |  |  | Reverse | CCTGGAGGAGAAGAGGAAAGAGA |  |
|  |  |  | Probe | CCTGTTTCCGTAGCCTCATGAGCTGTT |  |
| *SLC7A11* | Solute carrier family 7 (anionic amino acid transporter light chain, xc- system), member 11 | NM_014331.3 4q28-q32 | Forward | GGTTATTCTATGTTGCGTCT | 92 |
|  |  |  | Reverse | AATAACAGCTGGTAGAGGAG |  |
|  |  |  | Probe | TCCATGATTCATGTCCGCAAGCA |  |
| *SNAP25* | Synaptosomal-associated protein, 25 kDa | NM_003081.3 NM_130811.2 20p12-p11.2 | Forward | TAACCACCATTTCCCCTGTG | 100 |
|  |  |  | Reverse | TTTATCAGCTACTAAACATCTCAGCAA |  |
|  |  |  | Probe | TGCTTAATCTAGAGCTATGCACACCA |  |
| *TXNRD1* | Thioredoxin reductase 1 | NM_001093771.2 NM_001261445.1 NM_001261446.1 NM_003330.3 NM_182729.2 NM_182742.2 NM_182743.2 12q23-q24.1 | Forward | GTGGAGAAGTTTGGGGAAGAA | 138 |
|  |  |  | Reverse | CACAACACGTTCATTGTCTTTAGT |  |
|  |  |  | Probe | CGATTCCGTCAAGAGATAACAACAAATGT |  |

^*^ Gene symbols and localization according to HGNC; ^†^ RefSeq accession numbers for all transcript variants targeted by the probe; ^+^ assay design based on Vandesompele et al. 2002; ^++^ assay design according to Casagrandi et al. 2003.
