# Supplementary material for: Dual 3’Seq using deepSuperSAGE uncovers transcriptomes of interacting Salmonella enterica Typhimurium and human host cells
Source: BMC Genomics. 2015 Apr 19;16(1):323. doi: 10.1186/s12864-015-1489-1 (PMC4480994; doi:10.1186/s12864-015-1489-1)
Supplement: Additional file 5: Table S4. — Primer sequences used for qRT-PCR quantification of selected mRNAs from host and pathogen cells. [file 12864_2015_1489_MOESM5_ESM.docx]

| **Gene symbol^*^** | **Alias^*^** | **Accession number^†^ / localization^*^** | **Primer** | **Sequence** | **Ampl. length** | **Ct of NTC** |
| --- | --- | --- | --- | --- | --- | --- |
| *B2M^+^* | Beta-2-microglobulin | NM_004048.2 15q21-q22.2 | Forward | TGCTGTCTCCATGTTTGATGTATCTG | 150 | und. |
|  |  |  | Reverse | AGCTTTGAGTGCAAGAGATTGAAGAG |  |  |
| *GAPD^+^* | Glyceraldehyde-3-Phosphate Dehydrogenase | NM_001289746.1 NM_001289745.1 NM_001256799.2 NM_002046.5 12p13.31 | Forward | GTCATGGGTGTGAACCATGAGAAG | 146 | und. |
|  |  |  | Reverse | GGCATGGACTGTGGTCATGAG |  |  |
| *RPL13A^+^* | Ribosomal protein L13a | NM_001270491.1 NM_012423.3 NR_003932.2 NR_004844.1 NR_026712.1 NR_073024.1 19q13.3 | Forward | CTGGAGGAGAAGAGGAAAGAGAAAG | 128 | und. |
|  |  |  | Reverse | GGTCTTGAGGACCTCTGTGTATTTG |  |  |
| *DDX58* | DEAD (Asp-Glu-Ala-Asp) Box Polypeptide 58 | NM_014314.3 9p12 | Forward | ACCTGACGGGAGGTGATTGG | 195 | und. |
|  |  |  | Reverse | CAAGTCTTACATGGCAGCAGATGAG |  |  |
| *DHX58* | DEXH (Asp-Glu-X-His) Box Polypeptide 58 | NM_024119.2 17q21.2 | Forward | TGGCTTAGGCTCCCGCAATG | 169 | und. |
|  |  |  | Reverse | GGCCTTGGTAGGGAAGGAATG |  |  |
| *IFIH1* | Interferon Induced With Helicase C Domain 1 | NM_022168.3 2q24.2 | Forward | CTGCAGTGTGCTAGCCTGTTC | 218 | und. |
|  |  |  | Reverse | AGACAAGGCAAATCTAAGCCTTTGTG |  |  |
| *NAIP* | NLR Family, Apoptosis Inhibitory Protein | NM_004536.2 NM_022892.1 5q13.2 | Forward | GTTACTGAGCTTGCTTGTGGTAAC | 195 | und. |
|  |  |  | Reverse | TTCCAGGCCCACTTCCTAGAG |  |  |
| *NFKB1* | Nuclear Factor Of Kappa Light Polypeptide Gene Enhancer In B-Cells | NM_001165412.1 NM_003998.3 4q24 | Forward | GGGATGAGGTTGCTTACTAAGCTTTG | 148 | und. |
|  |  |  | Reverse | AAATGATGCTGTGGTCAGAAGGAATG |  |  |
| *NFKB2* | Nuclear Factor Of Kappa Light Polypeptide Gene Enhancer In B-Cells 2 | NM_001261403.2 NM_001288724.1 NM_002502.5 NM_001077494.3 10q24 | Forward | ACGTACCGACAGACAACCTCAC | 186 | und. |
|  |  |  | Reverse | CGTACGCACTGTCTTCCTTCAC |  |  |
| *NFKBIA* | NFKB Inhibitor, Alpha | NM_020529.2 14q13 | Forward | GGGACGAGAAAGATCATTGAAATTCTG | 238 | und. |
|  |  |  | Reverse | ACCATTTACAGGAGGGTAACACAAAC |  |  |
| *NFKBIB* | NFKB Inhibitor, Beta | NM_001243116.1 NR_040515.1 NM_002503.4 19q13.1 | Forward | GATGCTGGAGCTGACCTTGAC | 132 | und. |
|  |  |  | Reverse | CAGGAGAAGCTCCAGCACATC |  |  |
| *NFKBID* | NFKB Inhibitor, Delta | NM_139239.1 19q13.12 | Forward | CAGCGTATGCTAATGTTGCAAACC | 207 | und. |
|  |  |  | Reverse | GCAGGGTGTTTCACAGAAACAATC |  |  |
|  |  |  |  |  |  |  |
| *NFKBIE* | NFKB Inhibitor, Epsilon | NM_004556.2 6p21.1 | Forward | ATGAGGGATGGAGGGACAAGAG | 113 | 37.1 |
|  |  |  | Reverse | CCTCCCTGGGTTACCCTTATCC |  |  |
| *NLRC5* | NLR Family, CARD Domain Containing 5 | NM_032206.4 16q13 | Forward | GGCACTGCCAAGACAATAAGC | 154 | und. |
|  |  |  | Reverse | GATCAAGCCTGGCTTCGAGTG |  |  |
| *NLRP11* | NLR Family, Pyrin Domain Containing 11 | NM_145007.3 19q13.43 | Forward | ACCCAGTTTGATCTTTCTGTCTGAAAC | 111 | und. |
|  |  |  | Reverse | GGGTTGCCTAGATGCTGTATTTGAC |  |  |
| *NOD1* | Nucleotide-Binding Oligomerization Domain Containing 1 | NM_006092.2 7p15-p14 | Forward | CAAGCAGCTGAAGGGTGACTAAAC | 116 | und. |
|  |  |  | Reverse | CATGGAGCAGCCACCACAAG |  |  |
| *REL* | V-Rel Avian Reticuloendotheliosis Viral Oncogene Homolog | NM_001291746.1 NM_002908.3 2p13-p12 | Forward | CAAGGCGGGTGGATCACTTG | 166 | 37.1 |
|  |  |  | Reverse | GCTTCCTGGGTTCAAACAATTATTGTG |  |  |
| *RELA* | V-Rel Avian Reticuloendotheliosis Viral Oncogene Homolog A | NM_001243985.1 NM_001243984.1 NM_001145138.1 NM_021975.3 11q13 | Forward | GTCTGGGACTTCCTTGCTCTC | 180 | und. |
|  |  |  | Reverse | AGCCAGCTTGGCAACAGATTTATTAG |  |  |
| *RELB* | V-Rel Avian Reticuloendotheliosis Viral Oncogene Homolog B | NM_006509.3 19q13.32 | Forward | CATATTCAGCCTTGGCGAGAAGC | 157 | und. |
|  |  |  | Reverse | GAGGCAGCTGTAACAAGCTAGTG |  |  |
| *TICAM1* | Toll-Like Receptor Adaptor Molecule 1 | NM_182919.3 19p13.3 | Forward | CCTGTGGACAGTGGAAGATGAG | 165 | und. |
|  |  |  | Reverse | CCAAGACCCTTCACCCAGAAATAG |  |  |
| *TLR3* | Toll-Like Receptor 3 | NM_003265.2 4q35 | Forward | CAAGGTACATCATGCAGTTCAACAAG | 190 | und. |
|  |  |  | Reverse | GCAATTTATGACGAAAGGCACCTATC |  |  |
| *TLR4* | Toll-Like Receptor 4 | NM_138557.2 NM_003266.3 NM_138554.4 9q33.1 | Forward | TGCACATATCCCTATGTATCCCTATCAG | 245 | und. |
|  |  |  | Reverse | CCATCTGCGGTCTCAGAGATCC |  |  |
| *nagD* | NagD protein | 740304  741056 (-) | Forward | GTTAAACAAAATGCAGGCGCACTC | 166 | 37.1 |
|  |  |  | Reverse | AAATCCAGCTTGGGCGGAAC |  |  |
| *ndh* | NADH dehydrogenase | 1250596  1251900 (+) | Forward | CCGCGGTTCAATGATGATTGAAG | 138 | und. |
|  |  |  | Reverse | ACGAATGACGCGGTTAATACTGC |  |  |
| *rpoD* | RNA polymerase sigma-70 factor | 3397106  3398953 (+) | Forward | TGAAGCGAAAGTGCTGCGTATG | 117 | und. |
|  |  |  | Reverse | CTTCGCTTCGATCTGACGGATAC |  |  |
| *trmA* | tRNA (uracil-5)-methyltransferase | 4368074  4369174 (-) | Forward | GGTGCAGGCTTATCCACGTATTC | 131 | und. |
|  |  |  | Reverse | TGATGTGTATACGGGAACTGATCG |  |  |
| *bigA* | Hypothetical surface-exposed virulence protein BigA | 3650653  3656547 (+) | Forward | CCAGCGTACAGCGTCATTACAG | 167 | 36.4 |
|  |  |  | Reverse | TCGCTGATACCGTCTTCCTTCC |  |  |
| *gst* | Glutathione S-transferase | 1482592  1483197 (-) | Forward | GTCGCTTTCAGACGACCAGTG | 146 | und. |
|  |  |  | Reverse | TTTGCGACACGCTGCATATAAC |  |  |
| ***pefD*** | Plasmid-encoded fimbrial chaperone | 82902  83594 (+) | Forward | CCCGACGCCGTACTACTTTG | 140 | und. |
|  |  |  | Reverse | GTTCCGTTCAGCGACAGTTTC |  |  |
| ***psiA*** | Plasmid SOS inhibition | 35368  36105 (-) | Forward | TGAGGAGCATGATCTGGAAACG | 197 | 37 |
|  |  |  | Reverse | ATTGCGTTCTCCAGCTTGTTCG |  |  |
| *ptsN*[…] | Nitrogen regulatory IIA protein | 3507688  3508179 (+) | Forward | TTTGACGCCGAGGTGCTATTAC | 178 | und. |
|  |  |  | Reverse | TCGTCAAAGCCGGAATTGAACAG |  |  |
| ***repA*[…]** | DNA replication | 91053  91934 (-) | Forward | CGTAAACTGCACGGACTGAAAC | 182 | und. |
|  |  |  | Reverse | CGACATCATCATGCGCTCTTTC |  |  |
| *rpsO* | 30S ribosomal subunit protein S15 | 3472329  3472598 (-) | Forward | CCGGTTCTACCGATGTTCAGG | 196 | und. |
|  |  |  | Reverse | CAGACCCAGACGCTCAATCAG |  |  |
| *rpsT* | 30S ribosomal protein S20 | 52280  52543 (-) | Forward | CAAGCCGTCGCTCTATGATGC | 187 | 37 |
|  |  |  | Reverse | GTTGATCTGTGCAGTCAGGTTAGC |  |  |
| *ssaU*[…] | Type III secretion system protein | 1457283  1458341 (+) | Forward | ATCGCTGAACGCAACTGCATC | 115 | und. |
|  |  |  | Reverse | GCTGCAACGGGTTCAAATAACG |  |  |
| *sseJ* | Type III secretion system effector protein | 1678146  1679372 (+) | Forward | AATGGAGGCGGCCAGTAATATTG | 145 | und. |
|  |  |  | Reverse | CTTCCTGGGTTGGATGGACAAG |  |  |
| *ssrB* | Two-component response regulator | 1433019  1433657 (-) | Forward | AACGCTGACACGACCAATCATC | 207 | und. |
|  |  |  | Reverse | TTAACCTCATTCTTCGGGCACAG |  |  |
| *STM1015* | Phage DNA Replication Protein | 1063797  1064546 (+) | Forward | TCAGGTTATCGATCGCCGTCTC | 159 | und. |
|  |  |  | Reverse | TTTCCGGTAGCTTTCCCAGTC |  |  |
| *STM1029* | Hypothetical bacteriophage lysozyme | 1072226  1072726 (+) | Forward | ACTCCGCTCAACGGGATTATTTC | 110 | und. |
|  |  |  | Reverse | TTCCTGCATTACCGTGGCATTG |  |  |
| *STM2239* | Predicted bacteriophage protein | 2336900  2337268 (-) | Forward | GGCCTGATGAGCATTCGCTTC | 148 | 37 |
|  |  |  | Reverse | CAGCATTGAAAGGCAGCCATC |  |  |
| *wcaE* | Glycosyltransferase | 2192034  2192780 (-) | Forward | GGCGGCCCGTCTCTATAAAG | 171 | und. |
|  |  |  | Reverse | ATAGGATAATTGCGCCCAAAATCCTG |  |  |
| *wzx* | Hypothetical transmembrane transport protein | 2181676  2183154 (-) | Forward | AGTATATTCTCAGCCTGTGGCTACC | 188 | und. |
|  |  |  | Reverse | TAATTTCCATCACCAGCGCATTG |  |  |
| *ydiN* | Putative MFS-family transport protein | 1398221  1399440 (-) | Forward | GGGCGTAGCTAACTTTCTTATCCC | 112 | 37.1 |
|  |  |  | Reverse | TGGCGGTGATAAATGTCAGTAGTG |  |  |
| *ydjM* | Conserved hypothetical protein | 1358456  1359055 (-) | Forward | GTTTCCGCCTGCCTATTCTGG | 141 | und. |
|  |  |  | Reverse | CTGCGAAGACCAGCGTATGAC |  |  |
| *ygfE* | Cell division protein | 3242447  3242776 (+) | Forward | TGCCGCATTGAATATCAGCTATGAG | 122 | und. |
|  |  |  | Reverse | TGATCAAGCAACGCCTGTTCG |  |  |
| *yjjQ* | Conserved hypothetical regulatory protein | 4826700  4827425 (+) | Forward | GCATTAACCAAATTGCGGCGTTAC | 139 | und. |
|  |  |  | Reverse | TTCTCTGGCTCCCTGAGAACTG |  |  |

^*^ Gene symbols, alias and localization according to HGNC and Kroger et al. 2012 (prokaryotic locations comprise the respective start and end sites along with the encoding strand in brackets); ^†^ RefSeq accession numbers for all transcript variants targeted by the respective primer pair; ^+^ assay design based on Vandesompele et al. 2002; no template control (NTC) reactions with no measurable amplification after 40 cycles of qRT-PCR are designated as undetermined (und.); primers for prokaryotic transcripts were additionally validated with 40 ng of host cell total RNA as additional NTC (only the lowest Ct value for both NTC reactions is listed); additional coding sequences of polycistronic transcripts from the prokaryote are indicated by brackets and plasmid-encoded transcripts by bold gene symbols.
